# Diverse Misfolding Mutant Digestive Enzymes Cause Chronic Pancreatitis Through Common Pathways

**DOI:** 10.1016/j.jcmgh.2025.101638

**Published:** 2025-09-22

**Authors:** Steven J. Wilhelm, Grace E. Curry, Neel Matiwala, Jianguo Lin, Tran Quach, Mark.E. Lowe, Miklós Sahin-Tóth, Xunjun K. Xiao

**Affiliations:** 1 Department of Pediatrics, Washington University School of Medicine, St Louis, Missouri; 2 Department of Surgery, University of California Los Angeles, Los Angeles, California

**Keywords:** CPA1, ER Stress, Pancreatic Atrophy, PNLIP, Proteotoxicity

## Abstract

**Background & Aims:**

Increasing evidence suggests that protein misfolding and proteotoxicity is an important mechanism of chronic pancreatitis (CP) in patients with genetic variants. Two mouse models carrying misfolding digestive enzyme variants, *CPA1* N256K and *PNLIP* T221M, recapitulate the human CP phenotype. We hypothesized that both models develop CP through similar disease mechanisms.

**Methods:**

We conducted a comprehensive analysis of mice aged 1 to 6 months using histology, immunohistochemistry, protein immunoblotting, quantitative polymerase chain reaction (qPCR), transmission electron microscopy (TEM), and RNA sequencing (RNA-seq) analysis to characterize pancreatic pathological changes.

**Results:**

Both homozygous models exhibited CP hallmarks, including progressive acinar cell loss, inflammation, fibrosis, and fatty replacement. CP progression was slower and less severe in *Cpa1* N256K mice compared with *Pnlip* T221M mice, and heterozygous mice showed slower CP development than homozygotes. Both mutant proteins misfolded in the pancreas, inducing endoplasmic reticulum stress and activating the unfolded protein response. RNA-seq analysis revealed slight differences in altered pathways at 1 month, but these differences disappeared by 3 months. Notably, apoptosis pathways were among the top upregulated pathways, confirmed by qPCR and immunohistochemistry. Differential expression and pathway analyses indicated early activation of both intrinsic and extrinsic apoptosis pathways elicited through multiple mechanisms.

**Conclusions:**

Our study demonstrates that *Cpa1* N256K and *Pnlip* T221M mice develop CP through similar mechanisms with slight differences in progression and severity. Both models could serve as invaluable tools for developing and testing CP therapies. Targeting cell death pathways for therapy may be unfeasible given their redundancy. Instead, effective therapeutic strategies should focus on reducing the burden of misfolded digestive enzymes in the pancreas.

**Summary**

Protein misfolding is an important pathway for the pathogenesis of chronic pancreatitis. *Pnlip* T221M and *Cpa1* N256K mice develop the disease through similar mechanisms, making both models suitable for studying the generalized mechanism of chronic pancreatitis associated with protein misfolding.

Chronic pancreatitis (CP) is a debilitating and progressive inflammatory disease of the pancreas characterized by irreversible damage and dysfunction of the organ. Despite significant advancement in understanding its pathophysiology, effective treatment for CP remains elusive. The lack of therapeutic options stems largely from our incomplete understanding of the mechanisms driving the disease. The prevailing theory for CP pathogenesis involves organ autodigestion by aberrant intrapancreatic trypsin.^1^, ^2^, ^3^, ^4^, ^5^, ^6^, ^7^, ^8^, ^9^, ^10^, ^11^ Protein misfolding and proteotoxicity, the proposed mechanism in over 100 diseases, may also play a role in the pathogenesis of CP. In this pathway, accumulation of misfolded proteins in the endoplasmic reticulum (ER) induces ER stress and activation of the unfolded protein response (UPR), which attempts to restore cellular homeostasis.^12^^,^^13^ Prolonged or overwhelming ER stress can trigger maladaptive cellular responses, including inflammation and cell death.^12^^,^^13^ Increasing evidence from studies of genetic variants in *PRSS1*, *CTRC,* carboxypeptidase A1 (*CPA1*), *CEL*, and pancreatic triglyceride lipase (*PNLIP)* suggests that protein misfolding and ER stress-related proteotoxicity leads to CP.^14^, ^15^, ^16^, ^17^, ^18^, ^19^, ^20^, ^21^, ^22^, ^23^, ^24^, ^25^, ^26^, ^27^, ^28^

Two recently developed preclinical mouse models of protein-misfolding variants found in humans, *CPA1* p.N256K and *PNLIP* p.T221M, develop spontaneous CP with pathologic features that are similar to the human disease.^15^^,^^16^^,^^22^^,^^26^^,^^29^^,^^30^ The *CPA1* gene encodes carboxypeptidase A1, and the *PNLIP* gene encodes pancreatic triglyceride lipase. Although the reported disease course in the two mouse strains is similar, it remains unclear whether these unrelated enzymes carrying different misfolding variants drive CP through a common mechanism or via different pathways.

To address this knowledge gap, we conducted a comprehensive comparative analysis of the *Cpa1* N256K (named as *CPA1 N256K* in the original report) and *Pnlip* T221M mouse models. By identifying their commonalities and divergences, this parallel comparison validated the models’ suitability for studying the general disease mechanisms of CP associated with protein misfolding to facilitate the development of targeted therapies in the future.

## Results

### Pancreatic Atrophy in *Cpa1* N256K and *Pnlip* T221M Mice

**Figure 1:**
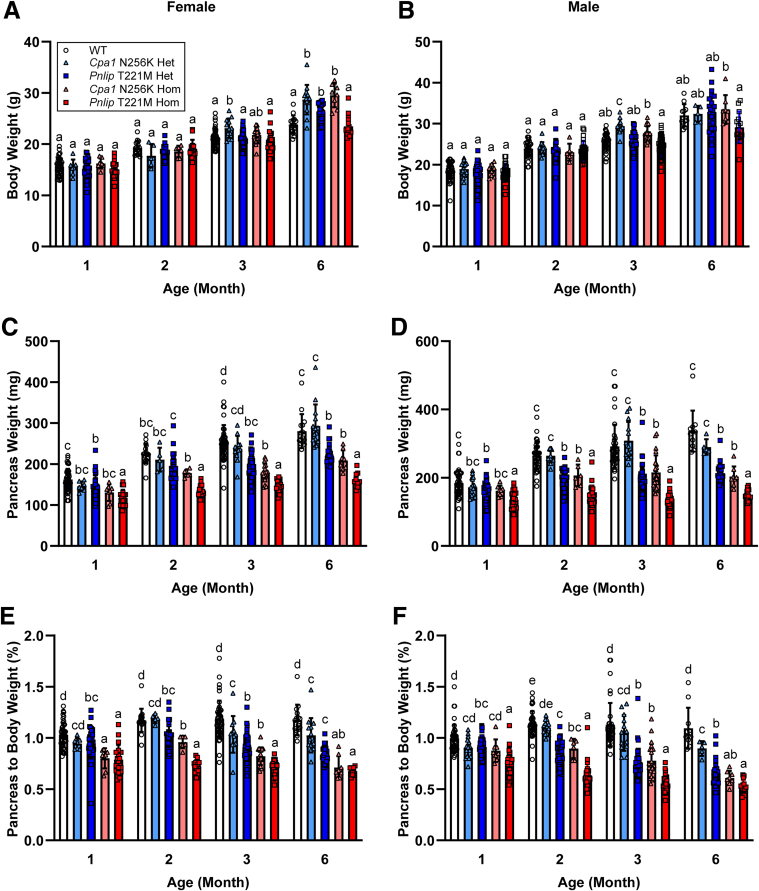
**Age-dependent changes in body weight and pancreas weight of *Cpa1* N256K and *Pnlip* T221M mice.** The data are presented as mean values with SD for each genotype at indicated age groups, with a sample size of n ≥ 8. Genotype groups include WT, *Cpa1* N256K heterozygous (Het) and homozygous (Hom), and *Pnlip* T221M Het and Hom. Body weight of females (*A*) and males (*B*). Pancreas weight of females (*C*) and males (*D*). Ratio of pancreas to body weight for females (*E*) and males (*F*). A 2-way ANOVA with multiple comparisons was performed at each indicated age point, separated by sex. Bars with entirely different letters (eg, “a” vs “b”) indicate a statistically significant difference between genotypes (*P* < .05), whereas bars with shared letters (eg, “a” vs “ab”) indicate no significant difference.

To evaluate the impact of CP on weight gain, we monitored the body weights of wild-type (WT) and of heterozygous and homozygous *Cpa1* N256K, and *Pnlip* T221M mice over a 6-month period. Across the 5 genotypes, the body weights of age- and sex-matched animals were generally comparable, with only a few significant but small differences observed between groups at 3 and 6 months of age (Figure 1*A and B*). In contrast, the pancreas weights of *Cpa1* N256K and *Pnlip* T221M homozygous mice were significantly lighter than those of age- and sex-matched WT mice, except in the *Cpa1* N256K female group at 2 months, where no difference was observed. The pancreas weights of *Cpa1* N256K heterozygous mice did not differ significantly from those of WT controls, whereas *Pnlip* T221M heterozygotes showed intermediate pancreas weights, except in the female group at 2 months (Figure 1*C and D*). When analyzing as the ratio of pancreas to body weight (Figures 1*E and F*), we found that the ratio for *Cpa1* N256K and *Pnlip* T221M homozygous mice were significantly lower than those for WT mice. Heterozygous mice exhibited intermediate ratios particularly by 3 months for females and 6 months for both sexes. These data confirm previous observations and indicate that *Cpa1* N256K and *Pnlip* T221M mice have pancreatic atrophy that progresses with age. The atrophy was more pronounced in homozygous mice compared with heterozygous mice, and more severe in *Pnlip* T221M mice relative to *Cpa1* N256K mice.

### Pancreas Histology in *Cpa1* N256K and *Pnlip* T221M Mice

**Figure 2:**
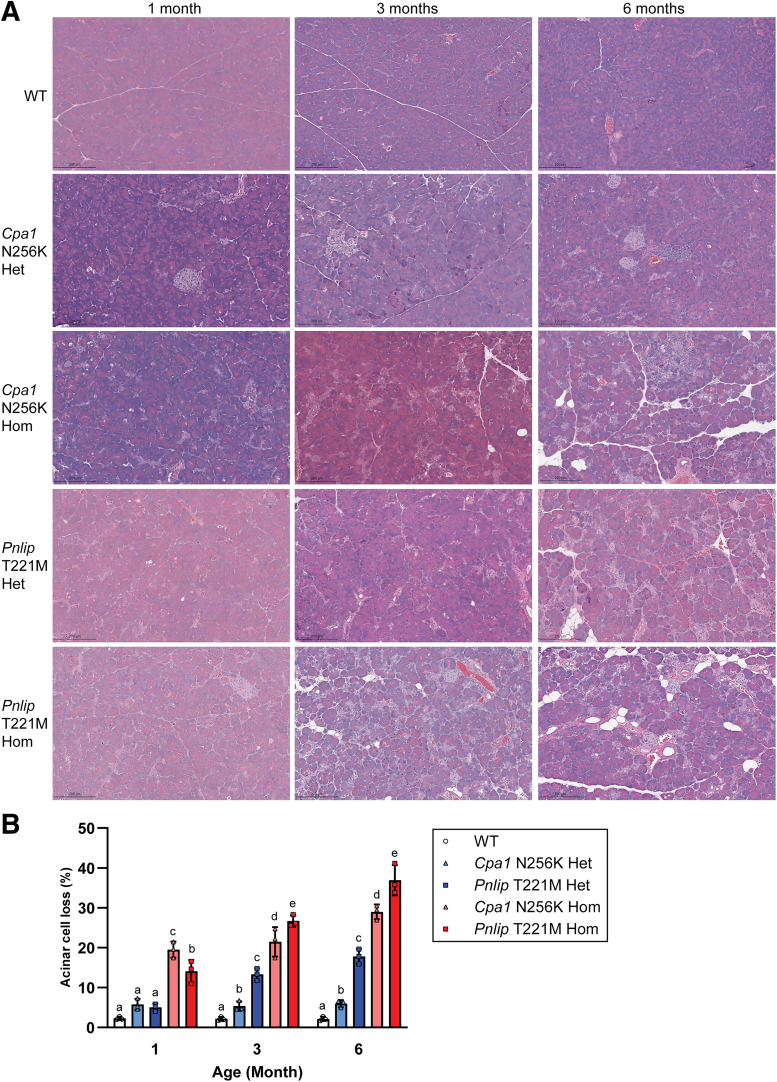
**Age-dependent histologic changes in the pancreas of *Cpa1* N256K and *Pnlip* T221M male mice.** The pathological changes were evaluated based on H&E staining. Genotype groups include WT, *Cpa1* N256K heterozygous (Het) and homozygous (Hom), and *Pnlip* T221M heterozygous (Het) and homozygous (Hom). (*A*) Representative H&E-stained images of the pancreas at 1, 3, and 6 months of age, showing the histological changes across different genotypes (n ≥ 6). Scale bar is 200 μm. (*B*) Quantification graphs of the percentage of pancreatic acinar cell loss at 1, 3, and 6 months of age. Data quantification was carried out as described in the Methods section. The quantification data are presented as mean values with SD for each genotype at the indicated age groups, with a sample size of n = 3. A 2-way ANOVA with multiple comparisons was performed at each indicated age point. Bars with entirely different letters (eg, “a” vs “b”) indicate a statistically significant difference between genotypes (*P* < .05), whereas bars with shared letters (eg, “a” vs “ab”) indicate no significant difference.

**Figure 3:**
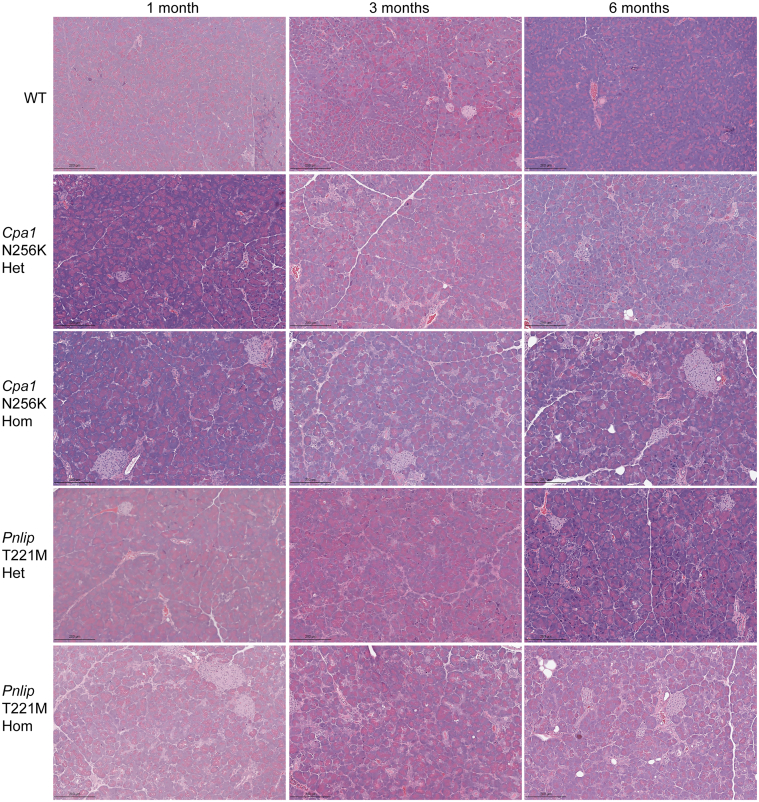
**Age-dependent pathologic changes in the pancreases of *Cpa1* N256K and *Pnlip* T221M female mice.** The pathological changes were evaluated based on H&E staining. Genotype groups include WT, *Cpa1* N256K heterozygous (Het) and homozygous (Hom), and *Pnlip* T221M heterozygous (Het) and homozygous (Hom). Representative H&E-stained images of the pancreas at 1, 3, and 6 months of age, showing the histological changes across different genotypes (n ≥ 6).

**Figure 4:**
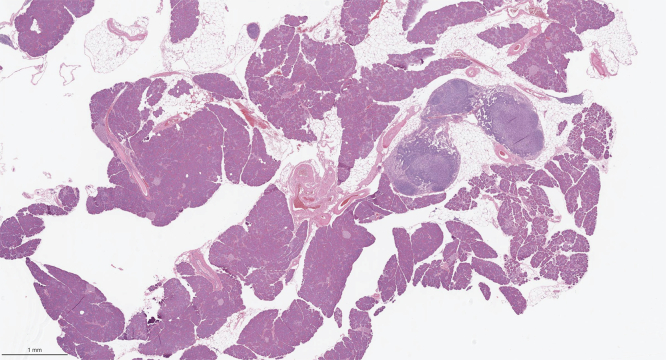
**Widespread pathologic changes in the pancreases of *Cpa1* N256K homozygous male mice at 6 months of age.** Representative image of H&E-stained mouse pancreas (n ≥ 6). The scale bar represents 1 mm.

**Figure 5:**
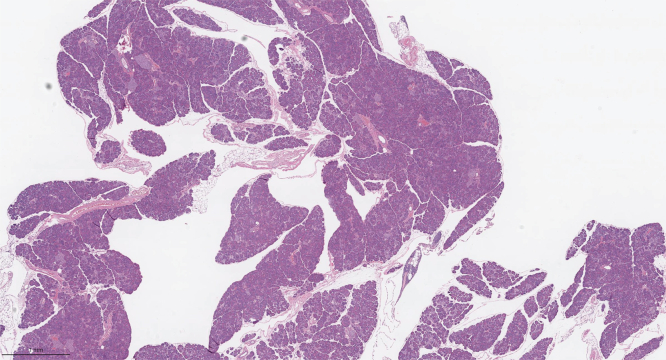
**Widespread pathologic changes in the pancreases of *Pnlip* T221M homozygous male mice at 6 months of age.** Representative image of H&E-stained mouse pancreas (n ≥ 6). The scale bar represents 1 mm.

Next, we compared the pathological changes in the pancreas of *Cpa1* N256K and *Pnlip* T221M mice by histology. In WT mice, hematoxylin and eosin (H&E) staining of pancreatic sections revealed the typical tightly packed acinar tissue with no discernible changes from 1 to 6 months of age (Figure 2*A* and Figure 3). In contrast, the pancreas of both *Cpa1* N256K and *Pnlip* T221M heterozygous and homozygous mice exhibited progressive pathological features indicative of CP, including acinar cell loss, fibrosis, infiltration of inflammatory cells, and increased intrapancreatic fat, although the progression was slower in heterozygous mice and the pancreatic phenotype was more pronounced in *Pnlip* T221M mice than in *Cpa1* N256K mice. Pathological changes were distributed throughout the entire pancreas in both mouse models (Figure 4 and Figure 5).

Given that both males and females of *Cpa1* N256K and *Pnlip* T221M mice developed similar pathological changes in the pancreas, we quantitated acinar cell loss only in male mice. Heterozygous and homozygous *Cpa1* N256K and *Pnlip* T221M mice demonstrated an age-dependent increase in pancreatic acinar cell loss (Figure 2*B*). Acinar cell loss was greater in homozygotes than in heterozygotes and in *Pnlip* T221M mice than in *Cpa1* N256K mice.

### Pancreatic Fibrosis and Immune Cell Infiltration in *Cpa1* N256K and *Pnlip* T221M Mice

**Figure 6:**
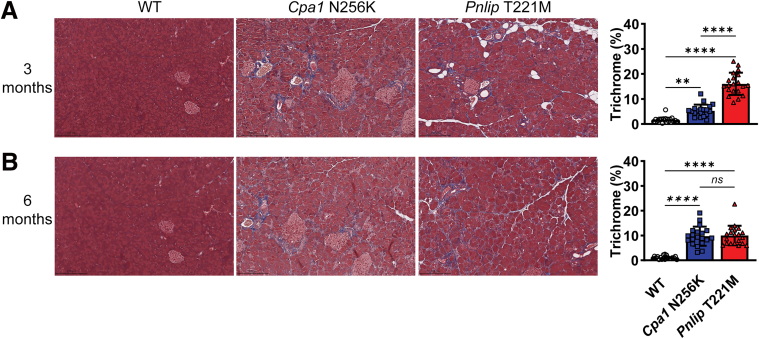
**Fibrosis in the pancreas of *Cpa1* N256K and *Pnlip* T221M male mice.** The mice analyzed are WT, *Cpa1* N256K and *Pnlip* T221M homozygous animals. Representative images of Masson trichrome blue stained pancreas sections, illustrating the extent of fibrosis in each genotype (n ≥ 5). (*A*) At 3 months of age. (*B*) At 6 months of age. Scale bar is 200 μm. The quantification graphs are presented on the right. Data quantification was carried out as described in the Methods section. The quantification data are presented as mean values with SD, with 20 fields randomly selected and analyzed for each group. One-way ANOVA with multiple comparisons was performed for each stain. Symbols ∗∗, ∗∗∗, and ∗∗∗∗ represent *P* < .01, .001, and .0001, respectively. *P* < .05 indicates a statistically significant difference between groups, and ns means not statistically significant.

**Figure 7:**
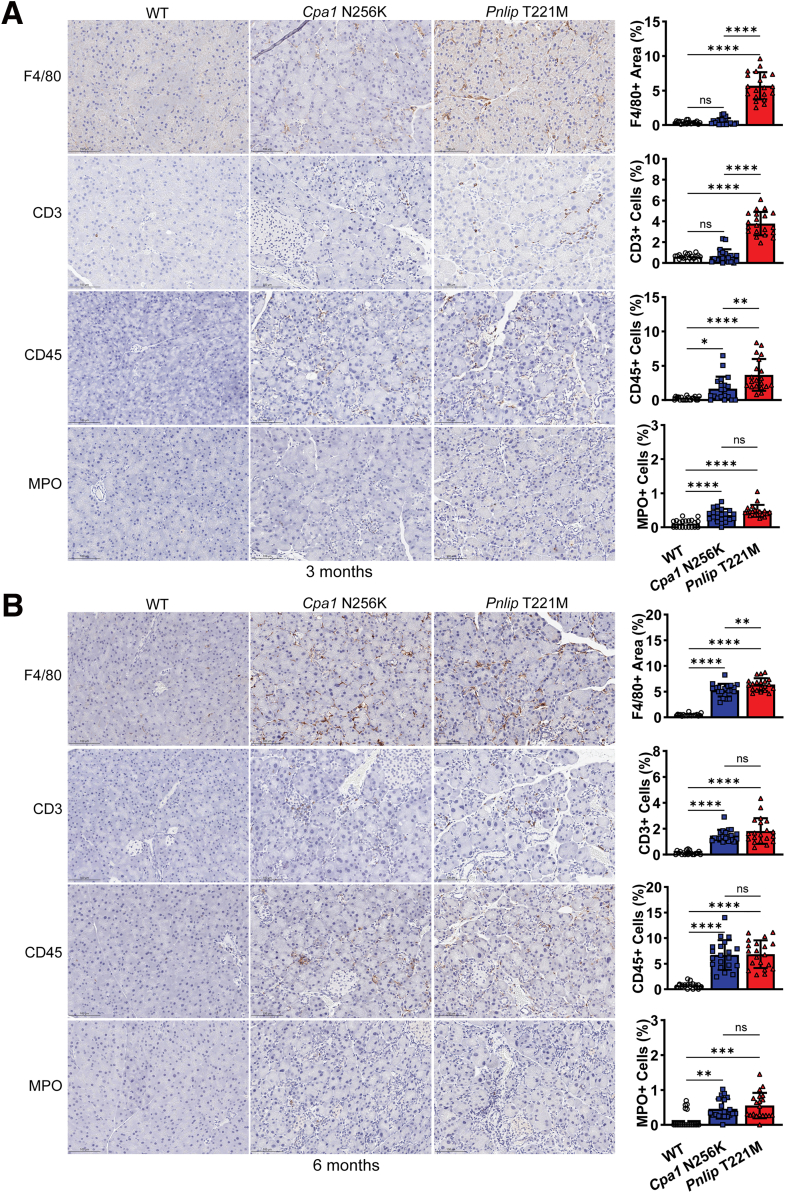
**Immune cell infiltration in the pancreas of *Cpa1* N256K and *Pnlip* T221M mice.** The mice analyzed are WT, *Cpa1* N256K, and *Pnlip* T221M homozygous animals. Representative immunohistochemistry images showing immune cell infiltration in the pancreas (n ≥ 5). (*A*) At 3 months of age. (*B*) At 6 months of age. The immune cell markers include: F4/80 for macrophages; CD3 and CD45 for T cells; MPO for neutrophils. Scale bar is 200 μm for F4/80 and 50 μm for CD3, CD45, and MPO. The quantification graphs are presented on the right for each stain. Data quantification was carried out as described in the Methods section. The quantification data are presented as mean values with SD, with 20 fields randomly selected and analyzed for each group. One-way ANOVA with multiple comparisons was performed for each stain. Symbols ∗, ∗∗, ∗∗∗, and ∗∗∗∗ represent *P* < .05, .01, .001, and .0001, respectively. *P* < .05 indicates a statistically significant difference between groups, and ns means not statistically significant.

We assessed pancreatic fibrosis in *Cpa1* N256K and *Pnlip* T221M homozygous mice by Masson’s trichrome staining. Compared to age-matched WT controls, both animal models exhibited a significant increase in staining at 3 and 6 months of age (Figure 6). Notably, fibrosis was more pronounced in *Pnlip* T221M mice at 3 months and was comparable between the two models at 6 months. We next evaluated pancreatic inflammation in these mice at 3 and 6 months via immunohistochemistry for markers of various immune cells (Figure 7). Macrophages (F4/80+ cells) were the predominant inflammatory cells. CD3+ lymphocytes (T-cells) and CD45+ cells (a pan-leukocyte marker) were less prominent. Neutrophils (MPO+ cells) were uncommon and when present tended to locate in areas of injury. Like fibrosis, inflammation occurred earlier in the *Pnlip* T221M mice than in *Cpa1* N256K mice. Both macrophages and T-cells were significantly increased in *Pnlip* T221M mice by 3 months. A similar increase was present in *Cpa1* N256K mice by 6 months. CD45+ lymphocytes and neutrophils were significantly increased in the pancreas of both mouse models at 3 and 6 months.

### Misfolding of Mutant Proteins in the Pancreas of *Cpa1* N256K and *Pnlip* T221M Mice

**Figure 8:**
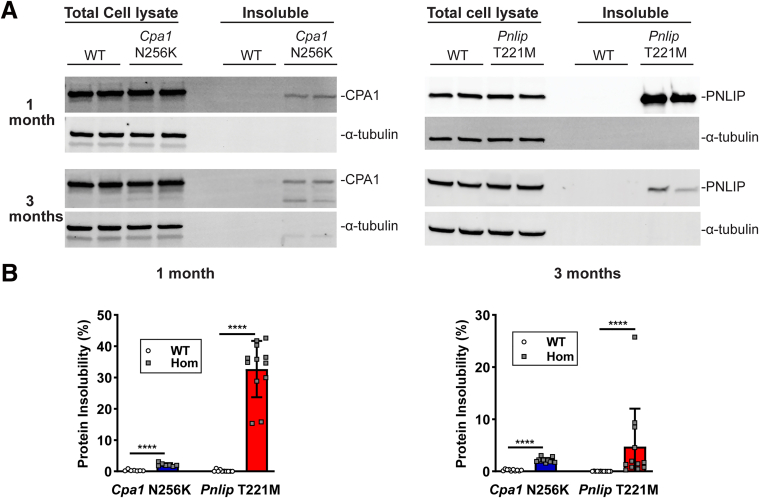
**Mutant protein misfolding in the pancreas of *Cpa1* N256K and *Pnlip* T221M mice.** The mice analyzed are WT, *Cpa1* N256K, and *Pnlip* T221M homozygous animals. (*A*) Representative immunoblots of CPA1 and PNLIP in the whole cell lysates and detergent-insoluble fractions of pancreatic protein extracts, and α-tubulin served as an endogenous control. Protein extraction and processing were performed on pancreas samples from mice at 1 and 3 months of age. (*B*) The quantification graphs of immunoblot analysis of CPA1 and PNLIP presented in *A*. The quantification data are presented as mean values with SD, with sample sizes of n ≥ 7 per genotype per age point. Multiple unpaired *t*-tests with Welch’s correction using the Holm-Sidak method were performed for each age group. Symbol ∗∗∗∗ represents *P* < .0001, with *P* < .05 indicating a statistically significant difference between groups.

To determine whether mutant protein misfolding occurs in the pancreas in these two mouse models, we fractionated protein extracts from the pancreas of 1- and 3-month-old mice into whole cell lysate and detergent-insoluble fractions for immunoblot (Figure 8*A*). CPA1 and PNLIP were present in the whole cell lysates from the pancreas regardless of genotype and age. Only the mutant forms of CPA1 and PNLIP were detectable in the detergent-insoluble fractions, suggesting they misfold. Protein insolubility expressed as the percentage of each protein in the insoluble form relative to its total amount in the whole cell lysate was significantly higher for CPA1 N256K and PNLIP T221M than for their WT counterparts (Figure 8*B*). The protein insolubility of CPA1 N256K remained low, approximately 1% to 2%, at both ages, whereas the protein insolubility of PNLIP T221M decreased from approximately 30% at 1 month to 5% at 3 months.

### Transcriptome Analysis of Cellular Pathways in the Pancreas of *Cpa1* N256K and *Pnlip* T221M Mice

**Figure 9:**
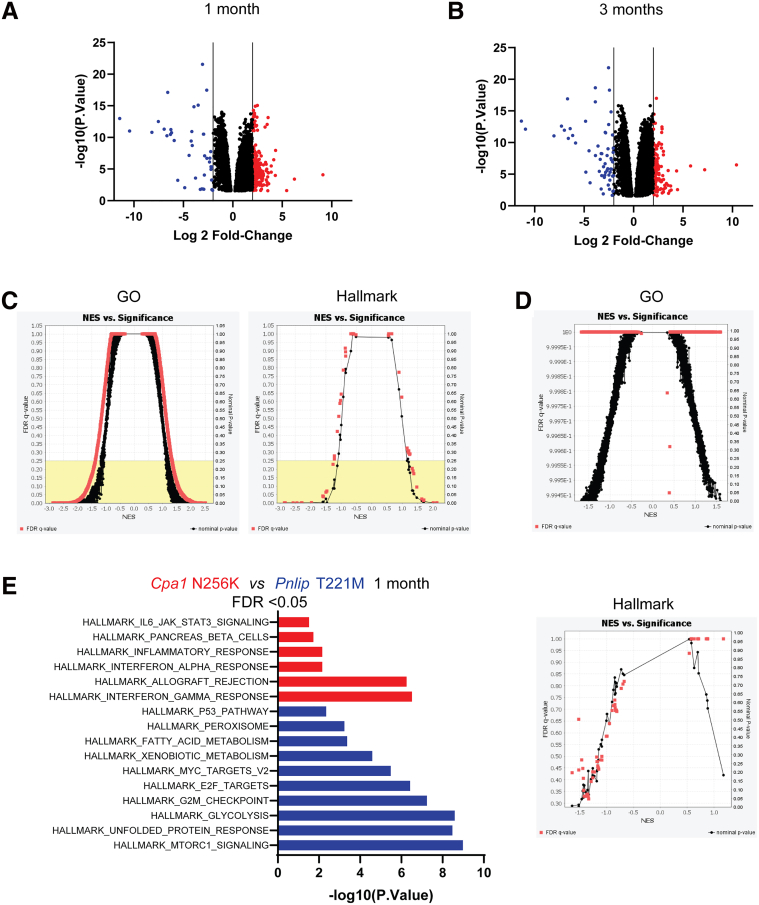
**Global comparison of transcriptomic profiles in the pancreas of *Cpa1* N256K and *Pnlip* T221M male mice.** Five *Cpa1* N256K and five *Pnlip* T221M homozygous male mice were analyzed by total RNA-seq as described in Methods at ages of 1 and 3 months. (*A*) Volcano plot of gene expression in the pancreas at 1 month. (*B*) Volcano plot of gene expression in the pancreas at 3 months. The plots show the fold change (FC) vs statistical significance of gene expression, with genes significantly downregulated (*blue*) and upregulated (*red*) in *Cpa1* N256K mice compared with *Pnlip* T221M mice at adjusted *P* < .05 and log2 FC >2.0. (*C*) Gene Set Enrichment Analysis (GSEA) of GO biological processes and Hallmark pathways at 1 month. (*D*) GSEA of GO biological processes and Hallmark pathways at 3 months. Plots display Normalized Enrichment Score (NES) vs significance of GSEA, with significantly enriched pathways in *Cpa1* N256K mice compared with *Pnlip* T221M mice at FDR <0.25. (*E*) Differentially regulated Hallmark pathways between the two strains at 1 month. *Red* and *blue* colors indicate pathways enriched in *Cpa1* N256K and *Pnlip* T221M mice at FDR <0.05, respectively. At 3 months, no GO biological processes or Hallmark gene sets reached significance at FDR <0.25.

To gain better understanding of the cellular pathways driving the onset and progression of CP in *Cpa1* N256K and *Pnlip* T221M mice, we analyzed RNA sequencing (RNA-seq) data on RNA isolated from the male mouse pancreas from both models and compared their transcriptomic profiles at 1 and 3 months of age. Relative to *Pnlip* T221M controls, volcano plots of 8429 genes with adjusted *P* value (*P*.adj) < .05 showed that 144 genes were upregulated (log2(fold change [FC]) >2.0) and 58 were downregulated (log2FC <−2.0) in *Cpa1* N256K mice at 1 month and that of 3284 genes, 53 genes were upregulated, and 38 genes were downregulated in *Cpa1* N256K mice at 3 months (Figure 9*A and B*). At 1 month, 1016 Gene Ontology (GO) biological process gene sets and 23 Hallmark gene sets were significantly enriched in *Cpa1* N256K mice at false discovery rate (FDR) <0.25 (Figure 9*C*). By 3 months, no GO biological process or Hallmark gene sets reached significance of FDR <0.25 (Figure 9*D*). Analysis of the Hallmark pathways at FDR <0.05 at 1 month showed 6 pathways were enriched in *Cpa1* N256K mice (Figure 9*E*). These pathways were mostly related to inflammation. Ten pathways, including the UPR and the P53 Pathway gene sets, were enriched in *Pnlip* T221M mice.

**Figure 10:**
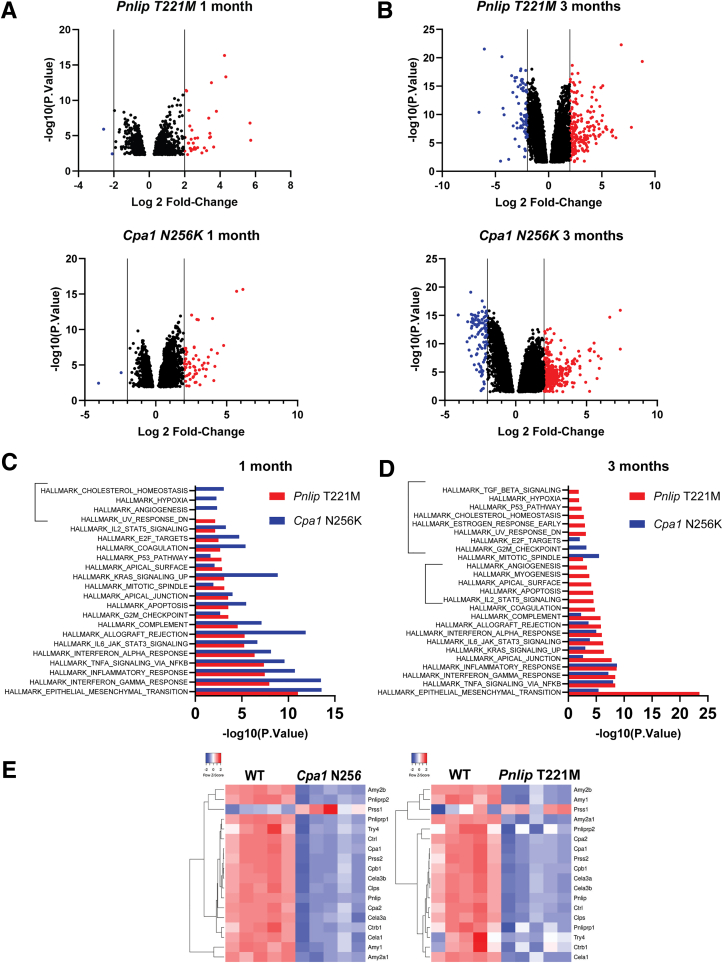
**Global comparison of transcriptomic profiles in the pancreas of *Cpa1* N256K and *Pnlip* T221M male mice vs WT controls.***Cpa1* N256K and *Pnlip* T221M homozygous and WT male mice were analyzed by total RNA-seq as described in Methods at ages of 1 and 3 months, with 5 animals per strain per age group. (*A*) Volcano plot of gene expression in the pancreas of *Pnlip* T221M and *Cpa1* N256K mice compared with WT controls at 1 month. (*B*) Volcano plot of gene expression in the pancreas of *Pnlip* T221M and *Cpa1* N256K mice compared with WT controls at 3 months. The plots show the fold change (FC) vs statistical significance of gene expression, with genes significantly downregulated (*blue*) and upregulated (*red*) in *Cpa1* N256K mice or *Pnlip* T221M mice compared with WT controls at adjusted *P* value < .05 and log2 FC >2.0. (*C*) Differentially regulated Hallmark pathways in the mutant mouse strains compared with WT controls at 1 month. (*D*) Differentially regulated Hallmark pathways in the mutant mouse strains compared with WT controls at 3 months. *Red* and *blue* colors indicate pathways enriched in *Pnlip* T221M mice and *Cpa1* N256K mice at FDR <0.05, respectively. (*E*) Heat map of the gene expression of selected pancreatic digestive enzymes in the mutant mouse strains vs WT controls at 3 months.

We next compared the transcriptomic profiles of *Cpa1* N256K and *Pnlip* T221M homozygous mice with WT controls. At 1 month, volcano plots showed that of 3523 genes with *P*.adj < .05, 59 were upregulated and 2 were downregulated in *Cpa1* N256K mice, and of 1355 genes, 35 were upregulated and 2 were downregulated in *Pnlip* T221M mice (Figure 10*A*). Pathway analysis of all genes enriched over WT (FDR <0.05) showed that the two mouse models had multiple pathways in common with only 4 enriched pathways not in common (Figure 10*C*). Even with a cutoff of FDR <0.25, there were still only 4 pathways that differed between *Cpa1* N256K and *Pnlip* T221M mice (Table 1).

**Table 1:** Hallmark Pathways Enriched in *Cpa1* N256K and *Pnlip* T221M Mice at 1 Month

Pathway	*Pnlip* T221M		*Cpa1* N256K	
	−Log10 *P* value	Log2FC	−Log10 *P* value	Log2FC
Common Hallmark pathways enriched at 1 month over WT FDR <0.25				
HALLMARK_EPITHELIAL_MESENCHYMAL_TRANSITION	11.03	6.95	13.57	7.80
HALLMARK_INTERFERON_GAMMA_RESPONSE	7.95	5.72	13.51	7.88
HALLMARK_INFLAMMATORY_RESPONSE	7.47	5.54	10.71	6.88
HALLMARK_TNFA_SIGNALING_VIA_NFKB	7.38	5.48	9.59	6.41
HALLMARK_INTERFERON_ALPHA_RESPONSE	6.38	5.11	8.13	5.93
HALLMARK_IL6_JAK_STAT3_SIGNALING	5.26	4.56	6.67	5.30
HALLMARK_ALLOGRAFT_REJECTION	5.30	4.49	11.87	7.28
HALLMARK_COMPLEMENT	4.56	4.09	7.12	5.37
HALLMARK_G2M_CHECKPOINT	3.55	3.48	2.67	2.88
HALLMARK_APOPTOSIS	3.54	3.48	5.47	4.58
HALLMARK_APICAL_JUNCTION	3.53	3.47	4.01	3.77
HALLMARK_MITOTIC_SPINDLE	3.12	3.20	1.94	2.29
HALLMARK_KRAS_SIGNALING_UP	3.11	3.19	8.87	6.12
HALLMARK_APICAL_SURFACE	2.88	3.12	2.07	2.44
HALLMARK_P53_PATHWAY	2.81	2.98	1.64	2.00
HALLMARK_COAGULATION	2.67	2.90	5.40	4.61
HALLMARK_E2F_TARGETS	2.49	2.74	4.73	4.18
HALLMARK_IL2_STAT5_SIGNALING	2.14	2.47	3.29	3.31
HALLMARK_ANGIOGENESIS	1.74	2.10	2.34	2.71
HALLMARK_HYPOXIA	1.66	2.07	2.27	2.57
HALLMARK_CHOLESTEROL_HOMEOSTASIS	1.51	1.88	3.06	3.19
HALLMARK_TGF_BETA_SIGNALING	1.47	1.83	1.22	1.34
Differing Hallmark pathways enriched at 1 month over WT FDR <0.25				
HALLMARK_UV_RESPONSE_DN	2.14	2.47	NS	NS
HALLMARK_MYOGENESIS	1.51	1.88	NS	NS
HALLMARK_ESTROGEN_RESPONSE_EARLY	1.07	1.37	NS	NS
HALLMARK_UV_RESPONSE_UP	NS	NS	1.22	1.56

FC, fold change; FDR, false discovery rate; WT, wild-type.

At 3 months, volcano plots showed that *Cpa1* N256K mice had 5879 differentially enriched genes with 361 significantly upregulated and 106 significantly downregulated compared with WT controls (Figure 10*B*). *Pnlip* T221M mice had 7285 differentially enriched genes with 227 significantly upregulated and 71 significantly downregulated. Ten Hallmark pathways were enriched in both mouse models, and 14 pathways were enriched in one or the other mouse model at FDR <0.05 (Figure 10*D*). Using a cutoff of FDR <0.25, 19 Hallmark pathways were enriched in both mouse models, and 10 pathways were only enriched in *Pnlip* T221M mice (Table 2). *Cpa1* N256K mice did not have unique enriched pathways.

**Table 2:** Hallmark Pathways Enriched in *Cpa1* N256K and *Pnlip* T221M Mice at 3 Months

PATHWAY	*Pnlip* T221M		*Cpa1* N256K	
	−Log10 *P* value	Log2FC	−Log10 *P* value	Log2FC
Common Hallmark pathways enriched at 3 months over WT FDR <0.25				
HALLMARK_EPITHELIAL_MESENCHYMAL_TRANSITION	23.50	10.89	5.41	4.58
HALLMARK_TNFA_SIGNALING_VIA_NFKB	8.40	6.05	8.02	5.75
HALLMARK_INTERFERON_GAMMA_RESPONSE	8.41	5.92	7.19	5.38
HALLMARK_INFLAMMATORY_RESPONSE	8.66	5.92	8.71	6.06
HALLMARK_APICAL_JUNCTION	7.77	5.65	2.63	2.85
HALLMARK_KRAS_SIGNALING_UP	6.37	5.11	3.04	3.14
HALLMARK_IL6_JAK_STAT3_SIGNALING	6.25	5.02	3.85	3.72
HALLMARK_INTERFERON_ALPHA_RESPONSE	6.02	4.97	5.02	4.39
HALLMARK_ALLOGRAFT_REJECTION	5.84	4.77	3.63	3.54
HALLMARK_COMPLEMENT	5.79	4.74	2.30	2.59
HALLMARK_COAGULATION	4.77	4.25	1.11	1.42
HALLMARK_IL2_STAT5_SIGNALING	4.52	4.06	1.70	2.06
HALLMARK_APOPTOSIS	4.45	4.03	1.10	1.42
HALLMARK_APICAL_SURFACE	4.11	4.02	2.63	2.85
HALLMARK_MYOGENESIS	3.78	3.63	1.03	1.32
HALLMARK_ANGIOGENESIS	3.35	3.53	1.63	2.03
HALLMARK_MITOTIC_SPINDLE	2.63	2.85	5.51	4.54
HALLMARK_G2M_CHECKPOINT	1.35	1.70	3.23	3.27
HALLMARK_E2F_TARGETS	0.96	1.23	2.06	2.39
Differing Hallmark pathways enriched at 3 months over WT FDR <0.25				
HALLMARK_UV_RESPONSE_DN	3.15	3.22	NS	NS
HALLMARK_ESTROGEN_RESPONSE_EARLY	2.99	3.10	NS	NS
HALLMARK_CHOLESTEROL_HOMEOSTASIS	2.79	3.00	NS	NS
HALLMARK_P53_PATHWAY	2.38	2.65	NS	NS
HALLMARK_HYPOXIA	1.93	2.28	NS	NS
HALLMARK_TGF_BETA_SIGNALING	1.87	2.25	NS	NS
HALLMARK_ESTROGEN_RESPONSE_LATE	1.40	1.76	NS	NS
HALLMARK_HEDGEHOG_SIGNALING	1.18	1.53	NS	NS
HALLMARK_WNT_BETA_CATENIN_SIGNALING	1.00	1.30	NS	NS
HALLMARK_NOTCH_SIGNALING	0.93	1.20	NS	NS

FC, fold change; FDR, false discovery rate; WT, wild-type.

Notably, the top common pathways enriched in both *Cpa1* N256K and *Pnlip* T221M mice at 1 and 3 months included those involved in fibrosis (epithelial-mesenchymal transition),^31^ inflammation, and the immune response (interferon gamma response, inflammatory response, tumor necrosis factor [TNF]-α signaling via nuclear factor kappa B [NF-κB], interferon alpha response, interleukin [IL]6 Janus kinase [JAK] Signal Transducer and Activator of Transcription 3 [STAT3] signaling, allograft rejection, and complement). At 1 month, pathways related to apoptosis, p53, G2M checkpoint, and Kras signaling up were among the top upregulated pathways in both mouse models. At 3 months, the apoptosis pathway and p53 pathway (FDR <0.05) were only enriched in the *Pnlip* T221M mice although the apoptosis pathway was enriched in both models with FDR <0.25 (Table 2). The expression levels of the essential executioners of necroptosis, *Ripk1*, *Ripk3*, and *Mlkl*, did not differ from control animals, suggesting that necrotic cell death was absent or minimal (data not shown).

At 1 month, few pathways were enriched in WT mice compared with either *Cpa1* N256K or *Pnlip* T221M mice (Table 3). The most enriched pathway in both comparisons was the oxidative phosphorylation pathway consistent with the mitochondrial dysfunction in the mouse models of CP. By 3 months, only oxidative phosphorylation and MYC targets V1 pathways were enriched in WT mice compared with *Pnlip* T221M mice. The same pathways as well as 12 other pathways were enriched in WT mice compared with *Cpa1* N256K mice. Because Hallmark pathways do not have a gene set for pancreatic digestive enzymes, we created a set and examined their expression pattern at 3 months. All but *Prss1* (T7 trypsinogen) were significantly downregulated in *Cpa1* N256K and *Pnlip* T221M mice, consistent with suppression of protein translation in reaction to stress (Figure 10*E*).

**Table 3:** Hallmark Pathways Enriched in WT Mice vs Mutant Mice

**PATHWAY**	*Pnlip* T221M		*Cpa1* N256K	
	−Log10 *P* value	Log2FC	−Log10 *P* value	Log2FC
Hallmark Pathways enriched at 1 month in WT animals FDR <0.05				
HALLMARK_OXIDATIVE_PHOSPHORYLATION	14.09	−8.07	4.50	−3.75
HALLMARK_MYC_TARGETS_V1	3.62	−3.53	NS	NS
HALLMARK_ADIPOGENESIS	3.21	−3.26	NS	NS
HALLMARK_KRAS_SIGNALING_DN	NS	NS	2.89	−3.23
Hallmark Pathways enriched at 3 months in WT animals FDR <0.05				
HALLMARK_OXIDATIVE_PHOSPHORYLATION	14.29	−8.11	24.33	−11.24
HALLMARK_MYC_TARGETS_V1	3.35	−3.36	9.46	−6.36
HALLMARK_ADIPOGENESIS	NS	NS	7.82	−5.66
HALLMARK_FATTY_ACID_METABOLISM	NS	NS	7.43	−5.54
HALLMARK_MTORC1_SIGNALING	NS	NS	5.72	−4.69
HALLMARK_HEME_METABOLISM	NS	NS	5.22	−4.45
HALLMARK_PROTEIN_SECRETION	NS	NS	4.08	−3.85
HALLMARK_XENOBIOTIC_METABOLISM	NS	NS	4.04	−3.79
HALLMARK_GLYCOLYSIS	NS	NS	3.73	−3.59
HALLMARK_DNA_REPAIR	NS	NS	3.66	−3.57
HALLMARK_PEROXISOME	NS	NS	3.11	−3.22
HALLMARK_UNFOLDED_PROTEIN_RESPONSE	NS	NS	2.98	−3.12
HALLMARK_ANDROGEN_RESPONSE	NS	NS	2.38	−2.66
HALLMARK_MYC_TARGETS_V2	NS	NS	1.88	−2.25

FC, fold change; FDR, false discovery rate; WT, wild-type.

**Figure 11:**
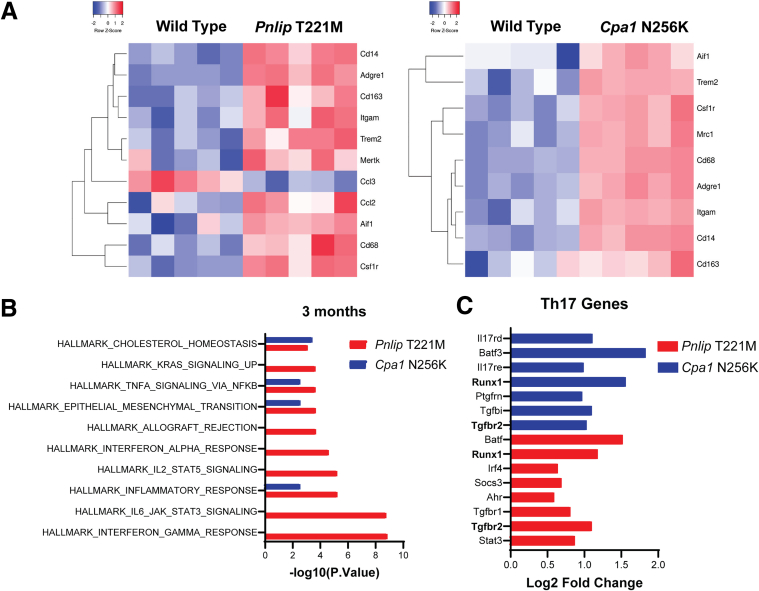
**Heat map of gene expression of key components involved in immune response in the pancreas of *Cpa1* N256K and *Pnlip* T221M homozygous mice vs WT controls.** Five *Cpa1* N256K and five *Pnlip* T221M homozygous male mice, along with five WT controls, were analyzed by total RNA-seq as described in the Methods, at 3 months of age. (*A*) Heat map of gene expression of major markers for immune cells in mutant mice vs WT controls. (*B*) Analysis of cytokine and chemokine expression in mutant mice vs WT controls revealing major pathways involved in the immune response. (*C*) Differential expression of genes involved in the Th17 response in mutant mice vs WT controls.

To further understand the immune response in our models, we analyzed the RNA-seq data from *Pnlip* T221M and *Cpa1* N256K mice at 3 months compared with WT controls to identify the populations of immune cells infiltrating the pancreas. Multiple markers of macrophages, including *Cd68* and *Cd163*, were upregulated in both models (Figure 11*A*). *Cd11c* was not present in either dataset, suggesting the *Cd68*^+^ macrophages are *Cd11c*^-^ as previously reported in human CP.^32^ T-cell marker *Cd4* was increased in *Pnlip* T221M (log2FC = 0.9; FDR = 0.01) but not detected in *Cpa1* N256K mice. These findings are consistent with the immunostaining for *Cd3*^+^ cells in Figure 7. *Cd8* was not detected in either model. Dendritic cell markers, *Itgax* (log2FC = 2.5; FDR = 2 × 10^-7^) and *Cd209a* (log2FC = 1.8; FDR = 5.5 × 10^-4^), were upregulated in *Pnlip* T221M mice. In contrast, they were downregulated in *Cpa1* N256K mice compared with WT animals. Monocyte markers *Cd14* and *Ccr2* were upregulated in both models (Figure 11*A*). Further analysis of macrophage subtypes showed that both cell types had increases in markers for M1 and M2 macrophages (Table 4). These findings are consistent with the complex immune environment reported in human CP.^32^^,^^33^

**Table 4:** Macrophage Subpopulations Enriched in Mutant Mice at 3 Months

		*Pnlip* T221M		*Cpa1* N256K	
Subpopulation	Gene	Log2FC	Adj *P* value	Log2FC	Adj *P* value
M1	Ccl2	0.7	.02	3.3	1.4 × 10^-8^
M1	Cd86	NS	NS	1.0	3.5 × 10^-4^
M1	Tlr2	0.9	5.6 × 10^-5^	1.1	1.1 × 10^-4^
M1	Nos2	1.7	8.4 × 10^-4^	NS	NS
M1	Cxcl10	1.7	.01	NS	NS
M2	Mrc1	1.8	9.5 × 10^-13^	1.8	3.5 × 10^-7^
M2	Cd163	0.7	.001	0.7	.03
M2	Retnla	1.7	1.7 × 10^-6^	1.0	.02
M2	Tgfb1	0.9	7.2 × 10^-8^	0.9	3.9 × 10^-6^

FC, fold-change.

We next used the RNA-seq data to analyze the cytokines and chemokines that were upregulated in the two CP models relative to WT controls. Multiple cytokines were upregulated in *Pnlip* T221M and *Cpa1* N256K mice, consistent with a heightened inflammatory state. Only a small subset of the two gene sets overlapped (Table 5). Pathway analysis revealed involvement of the IL-6/JAK/STAT3 and IL-2/JAK/STAT signaling pathways and showed an interferon gamma and alpha response in the *Pnlip* T221M mice (Figure 11*B*). These were not evident in the *Cpa1* N256K mice perhaps because of the apparent lag in progression of disease compared with *Pnlip* T221M mice. TNF alpha signaling through NF-κB and the inflammatory response were upregulated in both models. Examination of the data sets for components of the inflammasome revealed no upregulation of *Nlrp3*, *Pycard*, *Gsdmd*, *Casp1*, *Il1b*, or *Il18* in the canonical pathway nor upregulation of *Casp11*, *Tnf*, or *Tlr4* in the noncanonical pathway in *Pnlip* T221M mice. In the *Cpa1* N256K mice, only *Nlrp3* (log2FC = 2.7; FDR = 1.6 × 10^-4^) and Casp1 (log2FC = 0.9; FDR = 0.01) were significantly upregulated. This analysis suggests that inflammasome activation is not a prominent feature of the immune response in these models of CP.

**Table 5:** Upregulated mRNA Expression of Cytokines and Chemokines in Mutant Mice vs WT Mice at 3 Months

Gene	Pnlip T221M, (Log2FC)	Cpa1 N256K, (Log2FC)
Ildr2	1.031203	ND
C1qtnf7	1.075389	ND
Tnfaip3	1.179821	ND
Il2rb	1.082691	ND
Il3ra	1.027035	ND
Tnfrsf10b	1.453469	ND
Il18bp	1.044571	ND
Il34	1.100562	ND
Tnfrsf19	2.377817	ND
C1qtnf6	2.172228	ND
Il7	1.408686	ND
Tnfaip8l1	1.226116	ND
Ifnlr1	1.07417	ND
Il1rl2	1.068391	ND
Cxcl10	1.728224	ND
Neil3	1.91962	ND
Tnfrsf12a	1.844163	2.066901
Cxcl16	2.38074	2.383563
Cilp	4.829795	2.938295
Ccl8	3.696991	4.343186
Il1rn	4.301356	5.623889
Cxcl5	5.368345	5.375889
Ccl21d	ND	4.91503
Ccl17	ND	4.217626
Cxcl1	ND	3.983426
Lilr4b	ND	3.454413
Ccl2	ND	3.337921
Lilrb4a	ND	3.150241
Ccl28	ND	3.021273
Stil	ND	2.119972
Pilra	ND	2.259684
Tgfbr3l	ND	1.944735
Milr1	ND	1.838612
Zwilch	ND	1.769125

FC, fold change; WT, wild-type.

Because studies in human CP suggest that a Th17 response plays a central role in the pathophysiology of CP, we assessed whether a Th17 response was present in our CP models.^32^^,^^34^^,^^35^ The top upregulated Kyoto Encyclopedia of Genes and Genomes (KEGG) pathways include Th17 cell differentiation and IL-17 signaling pathways, but similar pathways did not appear in other pathway databases (Table 6 and 7). Examination of the RNA-seq data showed that 8 Th17-related genes were upregulated in *Pnlip* T221M mice, and 7 were upregulated in *Cpa1* N256K mice (Figure 11*C*). Two genes, *Runx1* and *Tgfbr2*, were upregulated in both models. These genes participate in Th17 differentiation, signaling, and cytokine responses, supporting a conclusion that Th17 pathways contribute to the immune response in both models. Overall, the findings show a robust inflammatory pattern involving both innate and adaptive immune pathways likely in response to injury and tissue remodeling. More careful, detailed analysis of the immune response will require single-cell RNA-seq analysis.

**Table 6:** Top 50 GO Biological Process Enriched in *Pnlip* T221M vs WT Mice at 1 Month

Pathway	Log2FC	−Log10 *P* value
GO:0002376 immune system process	10.11527888	23.30626
GO:0006955 immune response	9.615700383	20.98527
GO:0006952 defense response	9.128085334	19.07783
GO:0009605 response to external stimulus	8.498712463	16.85176
GO:0048583 regulation of response to stimulus	8.465398181	16.7968
GO:0048584 positive regulation of response to stimulus	8.456300953	16.688
GO:0007166 cell surface receptor signaling pathway	8.42428102	16.59109
GO:0002682 regulation of immune system process	8.402432578	16.38248
GO:0051707 response to other organism	8.314323279	16.03524
GO:0043207 response to external biotic stimulus	8.308640794	16.01561
GO:0007155 cell adhesion	8.295301919	15.99741
GO:0009607 response to biotic stimulus	8.279926809	15.92343
GO:0002684 positive regulation of immune system process	8.27096887	15.78948
GO:0022610 biological adhesion	8.2384677	15.79985
GO:0045087 innate immune response	8.220307437	15.48659
GO:0098542 defense response to other organism	8.187031428	15.48596
GO:0040011 locomotion	8.089812163	15.35135
GO:0044419 interspecies interaction between organisms	7.990574258	14.96251
GO:0001775 cell activation	7.956073663	14.76571
GO:0045321 leukocyte activation	7.91647702	14.60101
GO:0006928 movement of cell or subcellular component	7.910151152	14.75219
GO:0048869 cellular developmental process	7.863886677	14.65787
GO:0016477 cell migration	7.85171774	14.51137
GO:0048870 cell motility	7.836919305	14.47101
GO:0051674 localization of cell	7.836919305	14.47101
GO:0051239 regulation of multicellular organismal process	7.826708744	14.51357
GO:0030154 cell differentiation	7.821551972	14.51139
GO:0006950 response to stress	7.807510449	14.45898
GO:0002252 immune effector process	7.773554906	14.06753
GO:0050793 regulation of developmental process	7.547073711	13.56489
GO:2000026 regulation of multicellular organismal development	7.482955353	13.3328
GO:0051240 positive regulation of multicellular organismal process	7.437850217	13.17089
GO:0048513 animal organ development	7.425745487	13.18086
GO:0009653 anatomical structure morphogenesis	7.38981565	13.05181
GO:0006954 inflammatory response	7.228630234	12.30865
GO:0001816 cytokine production	7.201780988	12.27567
GO:0050776 regulation of immune response	7.157486589	12.10138
GO:0040012 regulation of locomotion	7.117256055	12.08837
GO:0001817 regulation of cytokine production	7.087629849	11.90242
GO:2000145 regulation of cell motility	7.042992331	11.85745
GO:0046649 lymphocyte activation	7.032739978	11.75977
GO:0030334 regulation of cell migration	6.974725617	11.64547
GO:0051270 regulation of cellular component movement	6.927614634	11.52193
GO:0002250 adaptive immune response	6.894665537	11.16639
GO:0002520 immune system development	6.861788882	11.31507
GO:0009966 regulation of signal transduction	6.740254221	11.04947
GO:0010604 positive regulation of macromolecule metabolic process	6.738125234	11.05241
GO:0051094 positive regulation of developmental process	6.733687676	10.98292
GO:0050778 positive regulation of immune response	6.698514748	10.68066
GO:0008283 cell population proliferation	6.64119873	10.73674

FC, fold change; GO, Gene Ontology; WT, wild-type.

**Table 7:** Top 50 GO Biological Process Enriched in Pnlip T221M vs WT Mice at 3 Months

Pathway	Log2FC	−Log10 *P* value
GO:0007155 cell adhesion	12.46674751	33.96658
GO:0022610 biological adhesion	12.40339101	33.65561
GO:0007166 cell surface receptor signaling pathway	12.24848673	33.41908
GO:0002376 immune system process	12.027854	32.27572
GO:0040011 locomotion	11.74477496	30.68194
GO:0006928 movement of cell or subcellular component	11.45253197	29.35067
GO:0030154 cell differentiation	11.39488445	29.33724
GO:0048583 regulation of response to stimulus	11.37908823	29.2636
GO:0009653 anatomical structure morphogenesis	11.3637193	29.05601
GO:0051239 regulation of multicellular organismal process	11.35924076	29.09909
GO:0016477 cell migration	11.34689114	28.67366
GO:0048513 animal organ development	11.25548125	28.60206
GO:0048869 cellular developmental process	11.24955911	28.63827
GO:0048870 cell motility	11.17462076	27.90658
GO:0051674 localization of cell	11.17462076	27.90658
GO:0006955 immune response	10.97529529	26.81248
GO:0009605 response to external stimulus	10.9721511	27.13608
GO:0050793 regulation of developmental process	10.82847241	26.5376
GO:0006952 defense response	10.55281643	24.95861
GO:2000026 regulation of multicellular organismal development	10.52738311	25.08407
GO:0048584 positive regulation of response to stimulus	10.27164041	23.95861
GO:0051240 positive regulation of multicellular organismal process	9.789008947	21.85078
GO:0009966 regulation of signal transduction	9.778685203	21.93181
GO:0040012 regulation of locomotion	9.748645201	21.451
GO:2000145 regulation of cell motility	9.612466425	20.88606
GO:0030334 regulation of cell migration	9.56278105	20.66959
GO:0002682 regulation of immune system process	9.551238937	20.74473
GO:0006950 response to stress	9.510151622	20.85699
GO:0051270 regulation of cellular component movement	9.437221076	20.22768
GO:0048468 cell development	9.406379516	20.33348
GO:0023051 regulation of signaling	9.402872369	20.40671
GO:0010646 regulation of cell communication	9.389629936	20.35164
GO:0009888 tissue development	9.31869878	19.93554
GO:0008283 cell population proliferation	9.156826018	19.32331
GO:0009607 response to biotic stimulus	9.134346092	19.07263
GO:0051707 response to other organism	9.127561683	19.03386
GO:0043207 response to external biotic stimulus	9.09660653	18.91721
GO:0030155 regulation of cell adhesion	9.08246367	18.66959
GO:0098542 defense response to other organism	9.063324657	18.64207
GO:0045595 regulation of cell differentiation	9.052447589	18.90309
GO:0030198 extracellular matrix organization	8.997798772	17.44977
GO:0043062 extracellular structure organization	8.997798772	17.44977
GO:0051094 positive regulation of developmental process	8.951640724	18.45469
GO:0001775 cell activation	8.879951088	18.04239
GO:0072359 circulatory system development	8.857283855	18.02045
GO:0042221 response to chemical	8.797375954	18.03245
GO:0044419 interspecies interaction between organisms	8.775434588	17.77211
GO:0042127 regulation of cell population proliferation	8.768696407	17.79317
GO:0002684 positive regulation of immune system process	8.761492024	17.53611
GO:0035295 tube development	8.714103488	17.48413

FC, fold change; GO, Gene Ontology; WT, wild-type.

**Figure 12:**
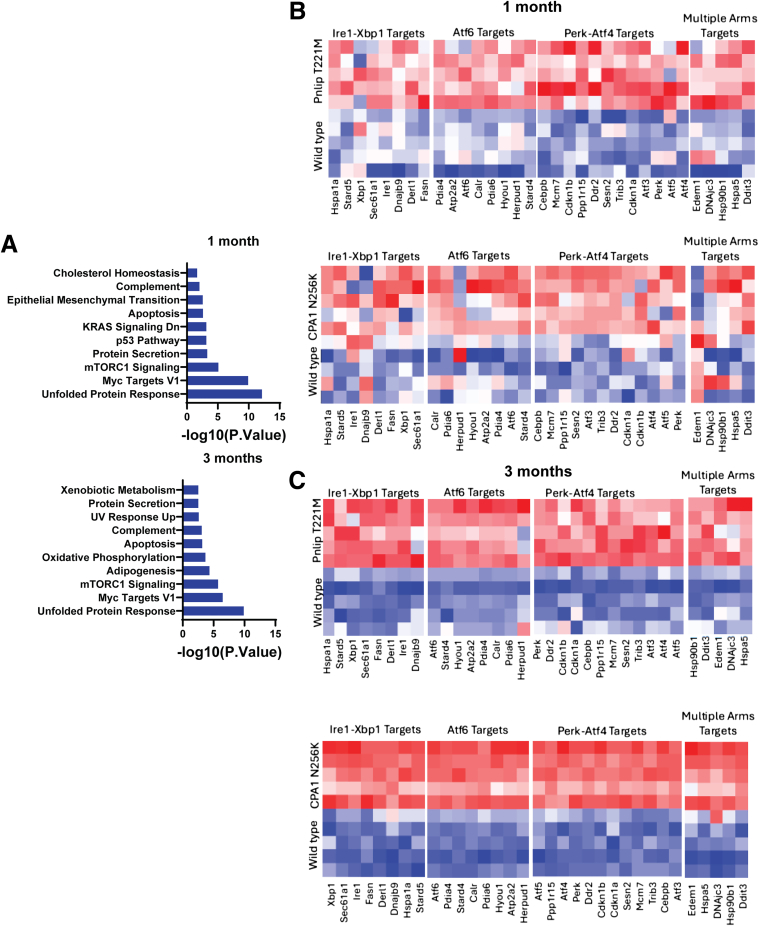
**Heat map of gene expression of key UPR pathway components in the pancreas of *Cpa1* N256K and *Pnlip* T221M homozygous mice vs WT controls.** Five *Cpa1* N256K and five *Pnlip* T221M homozygous male mice, along with five WT controls, were analyzed by total RNA-seq as described in the Methods, at 1 and 3 months of age. (*A*) Analysis of the top 300 expressed genes in the WT pancreas reveals the most enriched Hallmark pathways at 1 and 3 months. (*B*) Heat map showing gene expression of key downstream targets of the UPR pathways (Ire1, Perk, Atf6) in the pancreas of mutant mice compared with WT controls at 1 month. (*C*) Heat map showing gene expression of key downstream targets of the UPR pathways in the pancreas of mutant mice compared with WT controls at 3 months.

**Figure 13:**
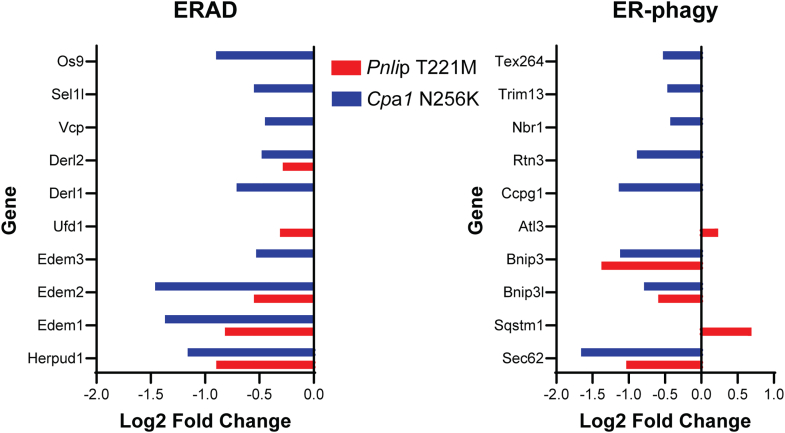
**Comparison of the expression of genes involved in the degradation of misfolded proteins in the pancreas of *Cpa1* N256K and *Pnlip* T221M mice vs WT controls.** Five *Cpa1* N256K and 5 *Pnlip* T221M homozygous male mice, along with five WT controls, were analyzed by total RNA-seq as described in the Methods, at 3 months of age. Two major degradation pathways involved in disposing of misfolded proteins are ERAD and ER-phagy. Key components of both pathways were analyzed and are presented here, showing that the majority of these genes are downregulated. *Red* and *blue* colors indicate genes in *Pnlip* T221M and *Cpa1* N256K mice, respectively.

Of note, the Hallmark UPR was not enriched at either 1 or 3 months even though there is evidence for misfolding of PNLIP T221M in vivo.^16^ To further investigate this unexpected finding, we analyzed a subset of the top 300 expressed genes in the WT pancreas at each age and performed enrichment analysis for Hallmark pathways (Figure 12*A*). The most significantly enriched Hallmark pathway at both ages was the UPR. We next investigated whether downstream targets of the IRE1, ATF6, and PERK arms of the UPR were upregulated. In both *Cpa1* N256K and *Pnlip* T221M mice, we found that gene targets associated with each arm of the UPR were upregulated at both 1 and 3 months, with the most pronounced differences at 3 months when compared with WT controls (Figure 12*B and C*). Genes that are targets of multiple arms of the UPR were also upregulated. The finding that downstream targets of the UPR are activated suggests the UPR is activated in the pancreas of both *Cpa1* N256K and *Pnlip* T221M mice. We also specifically analyzed genes related to ER-associated degradation (ERAD) and ER autophagy (ER-phagy) (Figure 13). In *Cpa1* N256K mice, the ERAD and ER-phagy components in the data set were all downregulated. In *Pnlip* T221M mice, all ERAD components were downregulated, and all ER-phagy genes were downregulated except for *Sqstm1* and *Atl3*. The downregulation of genes in these pathways is consistent with prolonged or chronic ER stress and a shift toward apoptosis. The upregulation of *Sqstm1* and *Atl3* may reflect distinct regulatory roles or context specific responses to ER stress.

**Figure 14:**
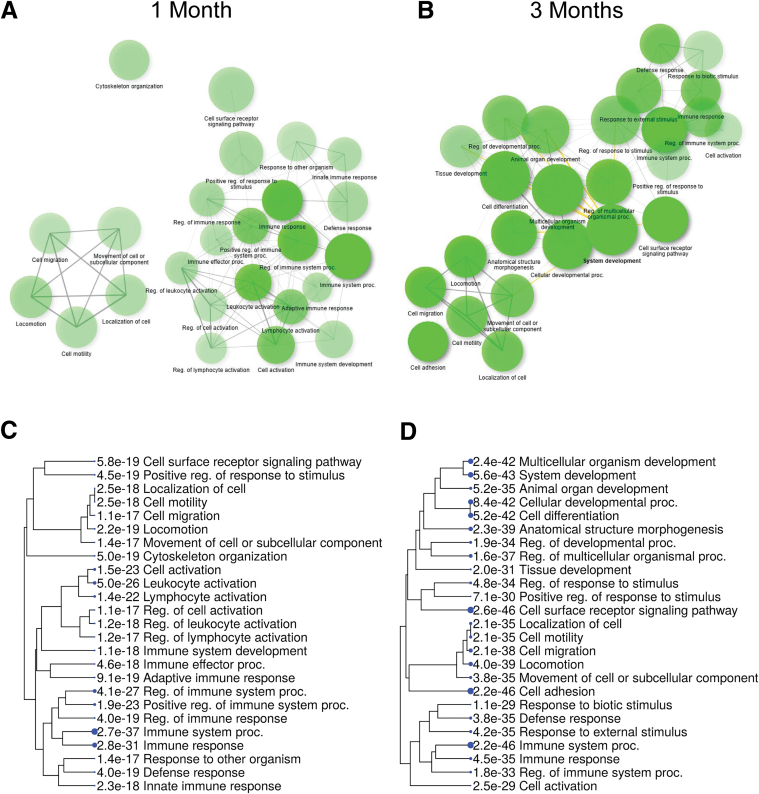
**Visualization of the relationship among the most enriched GO terms in the pancreas of *Pnlip* T221M mice.** Five *Pnlip* T221M homozygous and five WT male mice were analyzed by total RNA-seq as described in the Methods at 1 and 3 months of age, respectively. (*A*) Network analysis of the most 25 enriched GO terms at 1 month. (*B*) Network analysis of most enriched 25 GO terms at 3 months. Each cluster represents terms with a closer relationship. *Green nodes* indicate terms with significant enrichment, with darker green and larger sizes representing a higher degree of significance, as shown in the legend. (*C*) Hierarchical clustering tree of GO terms for biological processes at 1 month. (*D*) Hierarchical clustering tree of GO terms for biological processes at 3 months.

Because our analysis suggests that while the underlying mechanisms of disease onset may differ slightly, the pathways driving the onset and progression of CP in both models are highly similar, we focused on *Pnlip* T221M mice compared with WT mice to better define pathways and genes important for the development of CP in this model. We first analyzed GO Biological Process terms. GO:0048583 regulation of response to stimulus, GO:0009893 positive regulation of metabolic process, GO:0051234 establishment of localization, and GO:0006950 response to stress were the high-level GO terms with the largest number of genes at both ages (Tables 6 and 7). The 25 most enriched GO Biological terms showed an overlap of many terms at 1 and 3 months, but tree and network plots revealed an important difference (Figure 14). At 1 month, the terms separated into 2 clusters. The first cluster consisted of terms related to the immune response to a stimulus or stress, and the second cluster consisted of terms related to establishment of localization or cell movement. At 3 months, the clusters identified at 1 month were present. A third cluster enriched in terms related to development was present.

**Figure 15:**
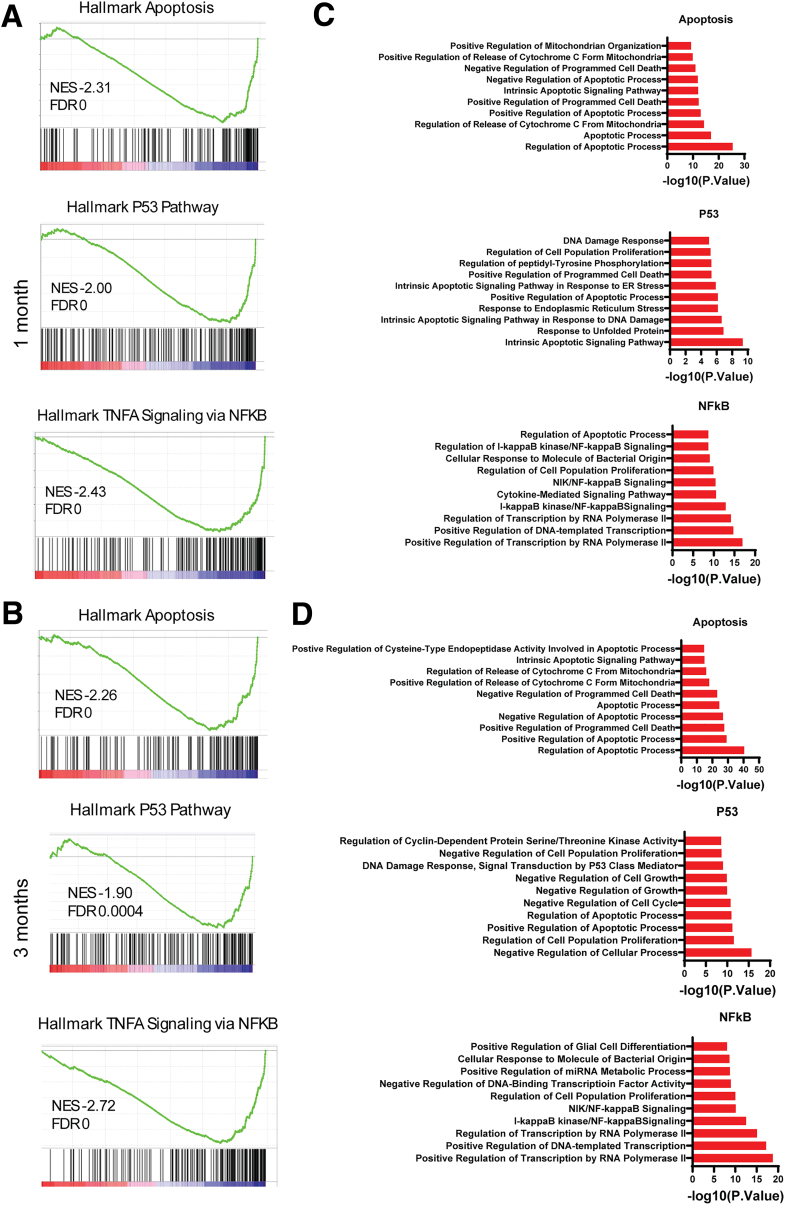
**Leading edge analysis of the selected enriched pathways in the pancreas of *Pnlip* T221M mice.** Five *Pnlip* T221M homozygous and five WT male mice were analyzed by total RNA-seq as described in the Methods at 1 and 3 months of age, respectively. GSEA demonstrated that the whole expressed genes were enriched in important pathways including Hallmark TNFα signaling via NF-κB, apoptosis, and P53 pathways. Leading edge analysis for each of these pathways generated an enrichment plot and identified a subset of genes that account for the enrichment signal. (*A*) Enrichment plots of the selected pathways at 1 month. (*B*) Enrichment plots of the selected pathways at 3 months. The Normalized Enrichment Score (NES) and FDRs were calculated for each gene set. Each *black bar* at the bottom of each panel represented a member gene of the respective gene set. GO enrichment analysis identified the biological processes that include the member genes in the subsets. (*C*) The important GO biological processes for each pathway at 1 month. (*D*) The important GO biological processes for each pathway at 3 months.

We next performed additional analysis of the enriched Hallmark Pathways. Because they likely have a significant role in the pathogenesis of CP, we concentrated TNFα signaling via NF-κB, apoptosis, and P53 pathways. Leading edge analysis for each pathway identified a subset of genes that account for the enrichment signal (Figure 15*A and B*; Supplementary Tables 1 [1 month] and 2 [3 months]). We then performed GO enrichment analysis to identify the biological processes that include the genes in the leading-edge subsets (Figure 15*C–D*). The analysis of the genes in the Hallmark apoptosis pathway at 1 and 3 months showed their products were active in similar biological processes related to the regulation of apoptosis. Of note, the intrinsic apoptosis pathway and regulation of release of cytochrome C from mitochondria were among the top 10 biological processes, suggesting the role of the intrinsic pathway and mitochondrial outer membrane permeabilization in the acinar cell loss observed in the *Pnlip* T221M mice. In contrast, the top 10 biological processes differed between 1 and 3 months in both the Hallmark P53 pathway and the TNFα signaling via NF-κB pathway. Although biological processes related to DNA damage and apoptosis were present in the P53 pathway at both ages, response to unfolded protein and response to ER stress were enriched at 1 month, whereas processes related to the cell cycle and cell growth were primarily enriched at 3 months. At 1 and 3 months, the top 10 biological processes for the gene subset of the TNFα signaling via NF-κB Pathway included processes related to transcription, signaling pathways and the immune response. Regulation of apoptotic process was among the top 10 listed at 1 month but not at 3 months.

**Figure 16:**
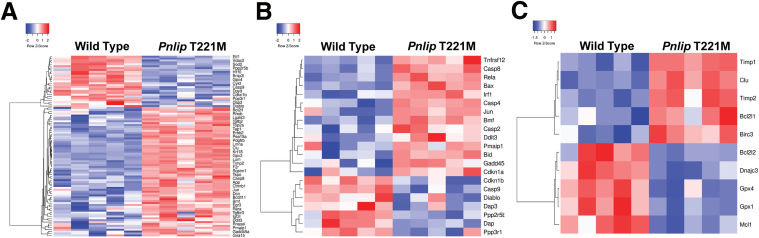
**Heat map of gene expression of key regulators of apoptosis in the pancreas of *Pnlip* T221M mice vs WT controls.** Five *Pnlip* T221M homozygous male mice and 5 WT controls at 3 months were analyzed by total RNA-seq as described in the Methods. (*A*) Heat map of differential expression of genes in the entire apoptotic gene set. (*B*) Heat map of differential expression of genes that are pro-apoptotic factors. (*C*) Heat map of differential expression of genes that are anti-apoptotic factors.

Because pathways that positively and negatively regulate apoptosis and cell death were prominent, we looked at the differential expression of genes in apoptotic pathways in our RNA-seq data set. Analysis of the entire apoptotic gene set revealed that the majority of genes were upregulated in *Pnlip* T221M mice, but there was a population of downregulated genes present (Figure 16*A*). Similarly, the majority of genes that positively regulate apoptosis were upregulated in *Pnlip* T221M mice (Figure 16*B*). The genes that negatively regulate apoptosis were evenly distributed by expression level (Figure 16*C*). Injury in the *Pnlip* T221M mice appears to trigger response involving upregulation of pro- and anti-apoptotic regulators, suggesting a dynamic balance between cell death and survival. Given the massive loss of acinar cells, the balance tilts toward cell death.

**Figure 17:**
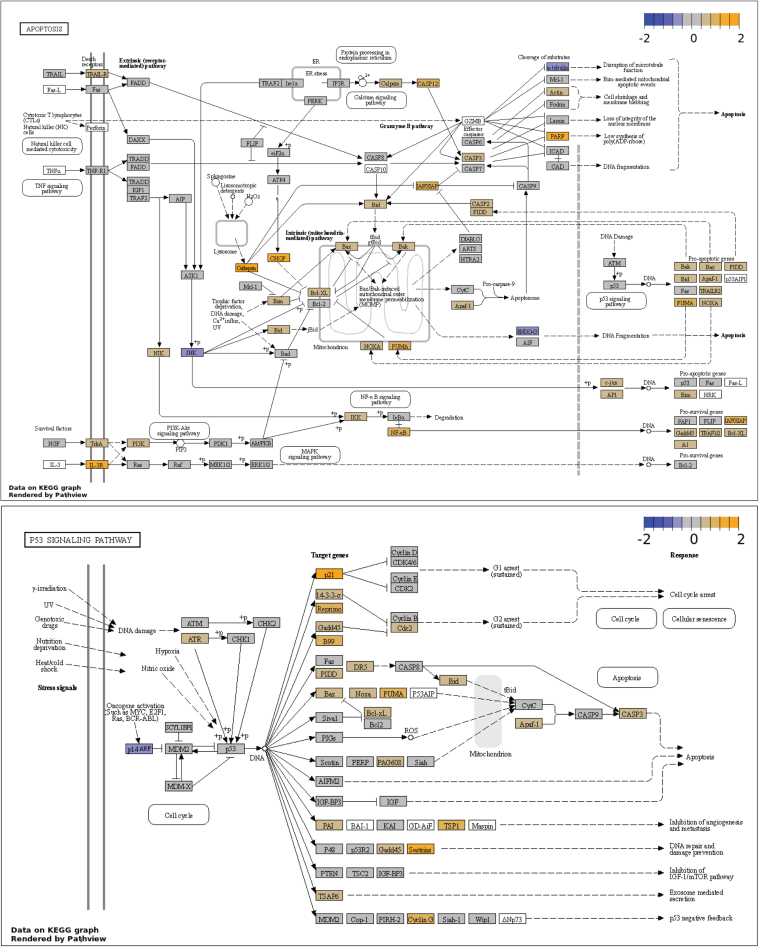
**Visualization of KEGG pathways for apoptosis and P53 signaling in the pancreas of *Pnlip* T221M mice.***Five* Pnlip T221M homozygous male mice and 5 WT controls at 1 month were analyzed by total RNA-seq as described in the Methods.The differently expressed genes compared with WT controls in each pathway was analyzed to map the graph.

To further analyze the processes and pathways that were activated early in the disease, we performed KEGG pathway enrichment analysis at 1 month.^36^^,^^37^ This time point is about 2 weeks after expression of *PNLIP* begins.^38^ Pathways involved in fibrosis, inflammation, and cellular signaling were enriched (FDR <0.05) (Table 8). We focused on the KEGG pathways for apoptosis (120 differentially expressed genes [DEGs]) and p53 signaling pathways (68 DEGs) (Figure 17). Multiple genes in the intrinsic apoptotic pathway were upregulated. Many of the upregulated genes are targets of the p53 pathway. In addition, genes in the extrinsic apoptotic pathway were upregulated, suggesting activation of apoptosis through death receptor 5 (*Tnfrf10b*). Examination of the p53 pathway also shows the upregulation of genes in the intrinsic apoptosis pathway as well as upregulation of genes leading to cell cycle arrest and DNA damage and repair.

**Table 8:** KEGG Pathways Enriched in *Pnlip* T221M vs WT Mice at 1 Month (FDR <0.05)

KEGG pathway	−Log10 *P* value	Log2FC
mmu04060 Cytokine-cytokine receptor interaction	6.94	5.31
mmu04380 Osteoclast differentiation	5.57	4.67
mmu04512 ECM-receptor interaction	4.36	4.05
mmu04145 Phagosome	4.35	3.98
mmu04514 Cell adhesion molecules	4.30	3.98
mmu04151 PI3K-Akt signaling pathway	4.39	3.97
mmu04668 TNF signaling pathway	4.25	3.94
mmu04612 Antigen processing and presentation	4.02	3.86
mmu04610 Complement and coagulation cascades	3.84	3.77
mmu04640 Hematopoietic cell lineage	3.86	3.74
mmu04010 MAPK signaling pathway	3.81	3.63
mmu04061 Viral protein interaction with cytokine and cytokine receptor	3.60	3.61
mmu04062 Chemokine signaling pathway	3.71	3.59
mmu04064 NF-kappa B signaling pathway	3.49	3.48
mmu04625 C-type lectin receptor signaling pathway	3.14	3.24
mmu04510 Focal adhesion	3.17	3.23
mmu04810 Regulation of actin cytoskeleton	3.10	3.18
mmu04621 NOD-like receptor signaling pathway	3.05	3.15
mmu04650 Natural killer cell mediated cytotoxicity	2.98	3.14
mmu04015 Rap1 signaling pathway	2.97	3.10
mmu04210 Apoptosis	2.90	3.06
mmu04659 Th17 cell differentiation	2.82	3.01
mmu04620 Toll-like receptor signaling pathway	2.81	3.01
mmu04670 Leukocyte transendothelial migration	2.73	2.94
mmu04657 IL-17 signaling pathway	2.63	2.89
mmu04672 Intestinal immune network for IgA production	2.53	2.87
mmu04115 p53 signaling pathway	2.59	2.84
mmu04530 Tight junction	2.56	2.80
mmu04630 JAK-STAT signaling pathway	2.46	2.73
mmu04360 Axon guidance	2.37	2.65
mmu04014 Ras signaling pathway	2.26	2.55

FC, fold change; KEGG, Kyoto Encyclopedia of Genes and Genomes; WT, wild-type.

### Confirmation of Activated Apoptotic Pathways in the Pancreas of *Cpa1* N256K and *Pnlip* T221M Mice

**Figure 18:**
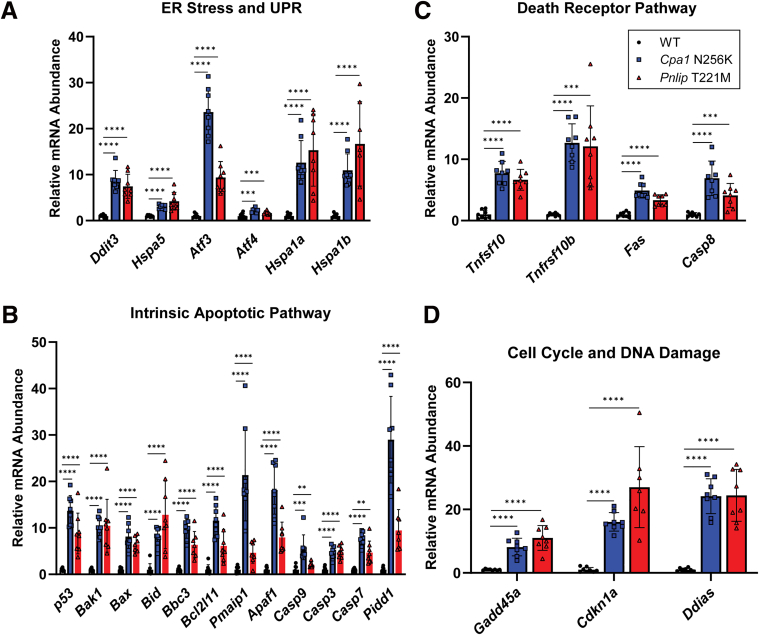
**Upregulation of key components of ER stress, UPR, and cell death pathways in the pancreas of *Cpa1* N256K and *Pnlip* T221M mice.** Pancreatic samples were collected from WT and *Cpa1* N256Kand *Pnlip* T221M homozygous male animals at 3 months of age and analyzed by qPCR. (*A*) ER stress and UPR. (*B*) Intrinsic apoptosis pathway. (*C*) Death receptor pathway. (*D*) Cell cycle and DNA damage. The quantification data are presented as mean values with SD, with at least 7 samples analyzed for each group. Multiple unpaired *t*-tests with the 2-stage step-up (Benjamini, Krieger, and Yekutieli) method were performed for each mutant mouse strain compared with WT. Symbols ∗, ∗∗, ∗∗∗, and ∗∗∗∗ represent *P* < .05, .01, .001, and .0001, respectively. *P* < .05 indicates a statistically significant difference between groups.

To confirm the upregulation of genes involved in ER stress, UPR, apoptosis, cell cycle regulation and DNA damage, we first employed quantitative polymerase chain reaction (qPCR) to quantitate major markers in these pathways. Multiple heat shock proteins (*Hspa5, Hspa1a, Hspa1b*) that help fold and stabilize proteins in the ER and downstream targets of the UPR (*Ddit3, Atf3, Atf4*) were significantly increased in both mouse models compared with WT controls (Figure 18*A*). Major components of the intrinsic apoptosis pathway were also upregulated in both mouse models (Figure 18*B*). These included many genes that are regulated by p53. In addition, the extrinsic apoptotic pathway was activated through death receptor 5 (*Tnfrsf10b*) (Figure 18*C*). Increased expression of *Gadd45a, Ddias*, and *Cdkn1a* (p21) were consistent with cell cycle regulation and DNA damage responses (Figure 18*D*). *Hspa1a* and *Hspa1b* also contribute to DNA repair, further indicating that DNA damage is part of the pathology in both mouse models.

**Figure 19:**
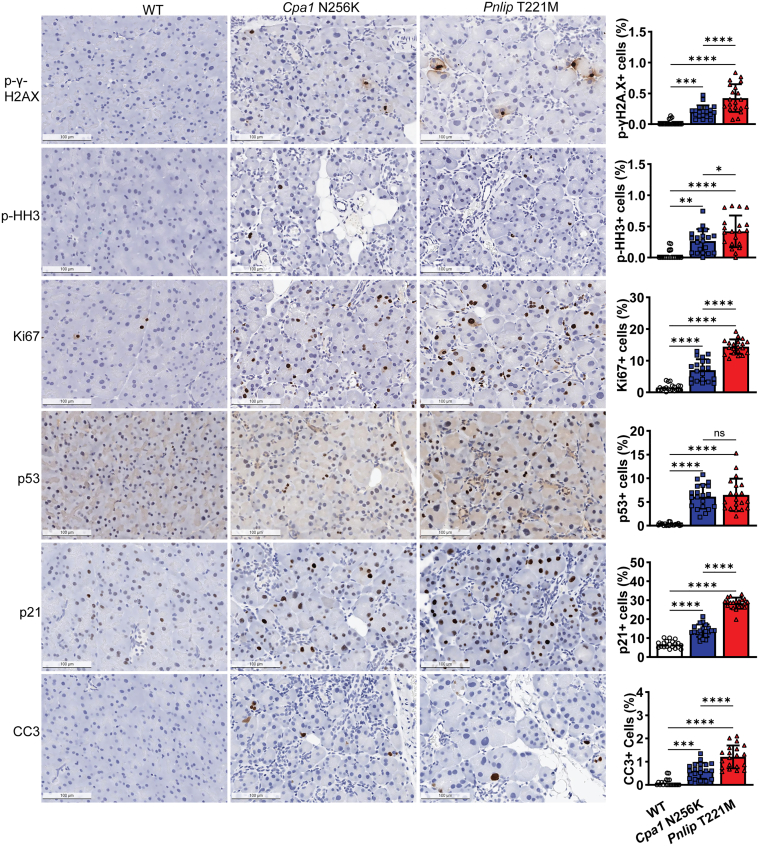
**Immunohistochemistry staining showing increased DNA damage and cell cycle markers in the pancreas of *Cpa1* N256K and *Pnlip* T221M mice.** The mice analyzed are WT, *Cpa1* N256K, and *Pnlip* T221M homozygous animals. Representative immunohistochemistry images showing increased DNA damage, cell proliferation, and apoptosis in the pancreas at 6 months of age. The markers include: phospho-histone H2A.X (p-γH2A.X), p-HH3, Ki67, p53, p21, and CC3. Scale bar is 100 μm. The quantification graphs are presented on the right for each stain. Data quantification was carried out as described in the Methods section. The quantification data are presented as mean values with SD, with 20 fields randomly selected and analyzed for each group. One-way ANOVA with multiple comparisons was performed for each stain. Symbols ∗, ∗∗, ∗∗∗, and ∗∗∗∗ represent *P* < .05, .01, .001, and .0001, respectively. *P* < .05 indicates a statistically significant difference between groups, and ns means not statistically significant.

Secondly, we used immunohistochemistry to further validate some of the critical findings obtained from our transcriptomic analysis (Figure 19). Compared with WT controls, both mouse models had increased staining for components in multiple pathways. A marker of DNA damage, p-γH2A.X, and markers of cell proliferation, p-HH3 or Ki67, were significantly increased. Consistent with our qPCR data, staining for p53 and p21(*Cdkn1a*) were increased. Lastly, we observed increased staining for cleaved caspase-3 (CC3), indicative of increased apoptosis. Notably, for each marker, the percentage of positively stained cells was relatively small although most staining was of acinar cells.

**Figure 20:**
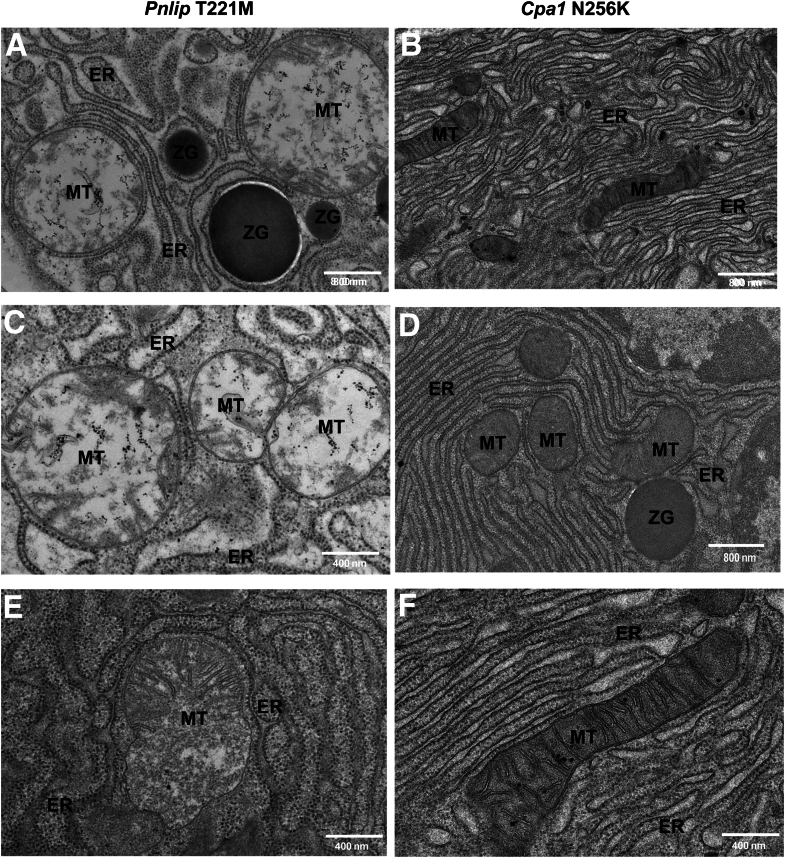
**TEM of the pancreatic acinar cells from mice.** TEM photomicrographs of the pancreas from homozygous *Pnlip* T221M (*A, C, and E*) and *Cpa1* N256K (*B, D, and F*) animals at 3 months are shown. The scale bars are shown in each subfigure. ER, endoplasmic reticulum; MT, mitochondria; ZG, zymogen granule.

We confirmed the activation of the mitochondrial apoptotic pathway by transmission electron microscopy (TEM) (Figure 20). Sections of mitochondria in *Pnlip* T221M acinar cells showed swollen mitochondria with intact cristae and double membranes as well as swollen mitochondria with remnants of cristae (Figure 20*A, C, and E*). Some of the swollen mitochondria had a ruptured outer membrane. These findings are consistent with the mitochondrial membrane permeability transition in apoptotic cells. In contrast, the mitochondria in *Cpa1* N256K acinar cells have a normal condensed morphology consistent with an excess of ADP (respiratory state III) (Figure 20*B, D, and F*).^39^^,^^40^

**Figure 21:**
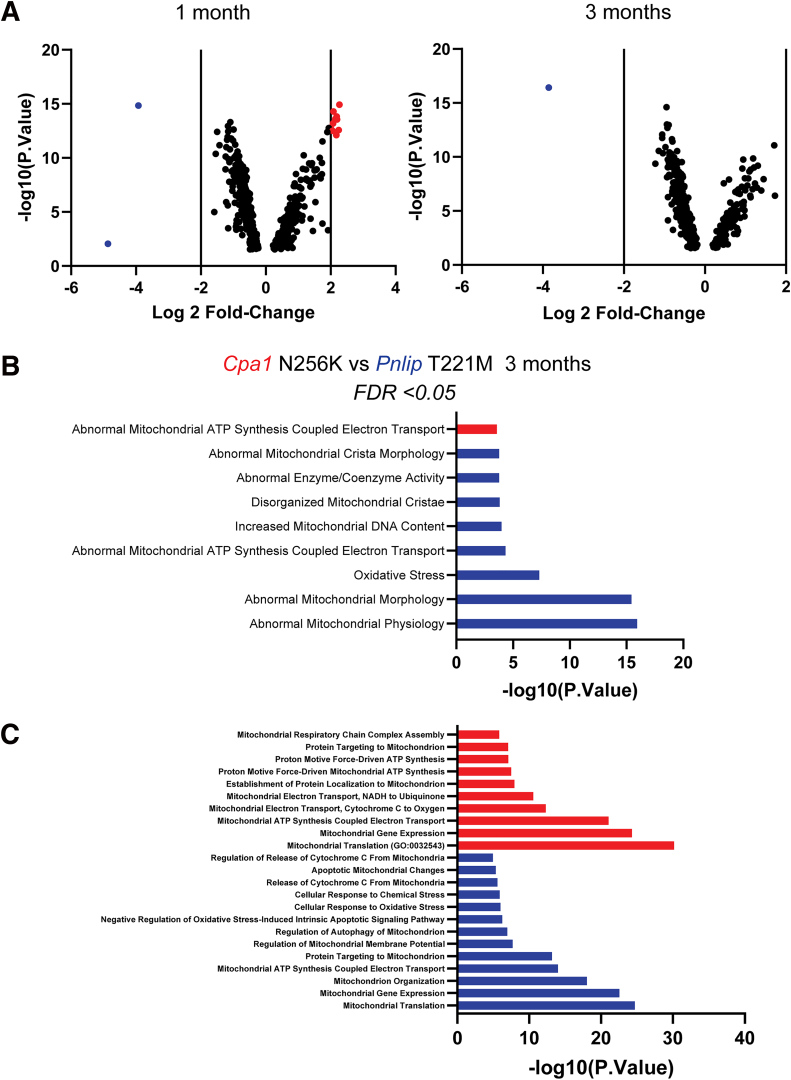
**Comparison of transcriptomic profiles of the mitochondria in the pancreas of *Cpa1* N256K vs *Pnlip* T221M male mice.** Five *Cpa1* N256K and 5 *Pnlip* T221M homozygous male mice were analyzed by total RNA-seq as described in Methods at ages of 1 and 3 months. (*A*) Volcano plots of gene expression in pancreatic mitochondria at 1 month and 3 months. The plots show fold change (FC) vs statistical significance of gene expression, with significantly downregulated genes (*blue*) and upregulated genes (*red*) in *Cpa1* N256K mice compared with *Pnlip* T221M mice at an adjusted *P* value < .05 and log2 FC >2.0. (*B*) Differentially regulated pathways in MGI Mammalian Phenotype Level 4 ontology database between the 2 strains at 3 months. (*C*) The pertinent pathways associated with mitochondria function in GO Biological Process enriched at 3 months. *Red* and *blue* colors in *B* and *C* indicate pathways enriched in *Cpa1* N256K and *Pnlip* T221M mice at FDR <0.05, respectively.

Given the differences in morphology of the mitochondria in the 2 mouse models, we further examined the transcriptome of the mitochondria. We created a list of the differentially expressed mitochondrial genes and differentially expressed nuclear-encoded genes included in a published list of 912 genes involved in mitochondria biogenesis and function.^41^ Volcano plots of 465 genes with *P*.adj < .05 show that 9 genes were upregulated (log2FC >2.0) and 2 were downregulated (log2FC <-2.0) in *Cpa1* N256K mice at 1 month, and that of 401 genes, no genes were upregulated, and 1 gene was downregulated in *Cpa1* N256K mice at 3 months (Figure 21*A*). *Lars2* was the only gene upregulated in *Pnlip* T221M mice at both 1 and 3 months. The genes upregulated in *Cpa1* N256K mice at 1 month were all mitochondrial genes except for *Dnajc15*. We then performed pathway analysis for the genes with FDR <0.05 at 3 months, the age analyzed by TEM (Supplementary Tables 3 [*Cpa1* N256K] and 4 [*Pnlip* T221M]). Only one pathway related to mitochondrial function from the MGI mammalian phenotype level 4 ontology database was upregulated in *Cpa1* N256K mice, abnormal mitochondrial ATP synthesis coupled electron transport MP:0010956. In contrast, 8 pathways were upregulated in *Pnlip* T221M mice (Figure 21*B*). These involved abnormal mitochondrial and mitochondrial crista morphology, disorganized mitochondrial crista, and oxidative stress. *Pink1* was common to all of these pathways.^42^ Increased expression of *Pink1* was confirmed by qPCR (2.70 ± 0.64 for *Pnlip* T221M group vs 1 ± 0.26 for WT group; *P* < .0001). GO biological process enrichment revealed 178 upregulated pathways in *Pnlip* T221M mice and 48 upregulated pathways in *Cpa1* N256K mice (Supplementary Tables 3 [*Cpa1* N256K] and 4 [*Pnlip* T221M]). The most pertinent pathways are shown in Figure 21*C*. Upregulated pathways in *Cpa1* N256K mice mainly involved ATP and energy production, whereas the upregulated pathways in *Pnlip* T221M mice involve pathways related to stress and mitochondrial apoptosis.

## Discussion

In this study, we aimed to comprehensively characterize the pancreatic phenotypes of two recently reported mouse models of CP associated with genetic variants that induce misfolding of the affected digestive enzyme. Both models were maintained on the same C57BL/6N genetic background and housed in the same facility to eliminate genetic and environmental variables that could influence our findings. The primary goal was to validate the common pancreatic phenotypic traits in these mouse models, both of which carry rare genetic variants linked to CP, and to establish their suitability for exploring the general mechanisms of CP related to protein misfolding.

Our most significant finding is that the pathophysiology of disease is similar in both models. This finding is supported by multiple observations. Histological methods show that both models have similar progressive CP phenotypes including acinar cell loss, fibrosis, and immune cell infiltration. Although the pathology is similar between the two models, we noted differences in disease progression and severity. Our results consistently show that the *Cpa1* N256K mice have less progressive and severe disease compared with the *Pnlip* T221M mice. The changes over time in various clinical parameters such as pancreas weight, the ratio of pancreas weight to body weight, pancreatic acinar cell loss, fibrosis, and immune cell infiltration support this observation.

Molecular methods confirm that common mechanisms drive CP in the two models with differences in progression and severity. Transcriptome analysis indicated that differentially expressed cellular pathways are similar in *Cpa1* N256K and *Pnlip* T221M mice. Comparison of the two models at 1 month identified a small number of Hallmark pathways that are differentially regulated in the two models. Inflammatory pathways were upregulated in *Cpa1* N256K mice, whereas pathways related to cell metabolism, UPR, and DNA damage were upregulated in *Pnlip* T221M mice. By 3 months, these differences vanished, and there were no significantly upregulated Hallmark pathways or GO biological process gene sets between the mouse models. The differences in pathway analysis at 1 month may be explained by differences in the kinetics of the disease process.

When the transcriptomes of the two mouse models of CP were compared with WT mice, Hallmark pathways involved in fibrosis, inflammation, immune response, and cell death were activated in both models. Noticeably absent were pathways related to ER stress and the UPR. Our analysis of PNLIP T221M and CPA1 N256K proteins in the pancreas showed that each was present in the detergent insoluble fraction, indicating misfolding of the protein variants; previous studies of these proteins and mouse models suggested that ER stress and the UPR were activated by expression of the protein variants.^16^^,^^22^ To further examine this unexpected finding, we performed enrichment analysis for Hallmark pathways in the WT mouse pancreas and found that the UPR pathway was the most significantly enriched pathway at both 1 and 3 months. We then looked at the expression levels of specific gene targets for the IRE1, ATF6, and PERK arms of the UPR. At 1 and 3 months, the expression of the gene targets was increased in both models with the clearest distinction at 3 months. Furthermore, we confirmed the upregulation of a subset of these targets by qPCR. These findings indicate activation of the UPR in both *Pnlip* T221M and *Cpa1* N256k mice. The lack of a significant enrichment in the Hallmark UPR pathway likely results from the high baseline expression of genes in this pathway in the WT pancreas and the asynchronous cell injury and death that occurs in vivo, which limits the sensitivity for detecting significant differences in the UPR through pathway analysis.

Analysis of the leading-edge genes in the Hallmark apoptosis, P53, and TNFα signaling via NF-κB pathways in *Pnlip* T221M mice confirmed the importance of ER stress and the UPR in triggering cell death through apoptosis. At 1 month, leading edge genes in each pathway were components in GO biological processes for ER stress and apoptotic pathways. By 3 months, the Hallmark apoptosis leading-edge genes were primarily components in apoptotic processes, whereas the leading-edge genes for the Hallmark P53 and TNFα signaling via NF-κB pathways contained components regulating DNA damage, cell cycle, cell differentiation, and cell growth. Cluster analysis of the top GO biological process gene sets confirmed that gene sets in the immune response to a stimulus or stress, establishment of localization or cell movement and development predominated. Through qPCR, we confirmed that components of the intrinsic apoptotic pathway, death receptor pathway, and cell cycle and DNA damage pathways were upregulated in both mouse models. Our findings suggest that cell death likely occurs through apoptosis triggered by persistent ER stress and a maladaptive UPR.

**Figure 22:**
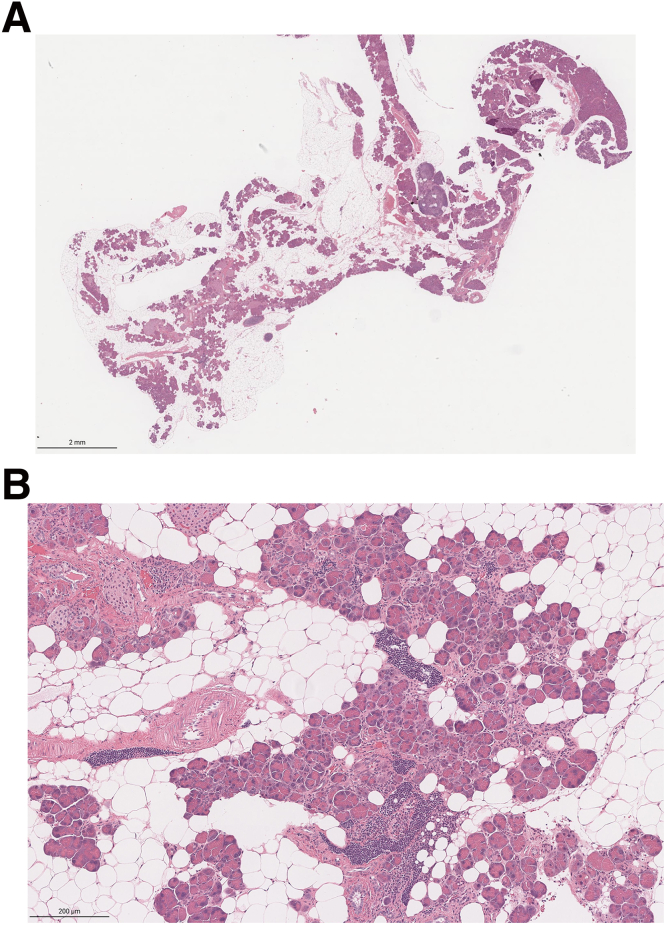
**Presence of visibly normal acinar cells in the pancreas of aged *Pnlip* T221M mice.** A representative image of the H&E-stained pancreas from a *Pnlip* T221M male mouse aged 20 months. The mouse body weight was 37 grams, pancreas weight was 178 milligrams, with a ratio of pancreas weight to body weight of 0.48%. Despite widespread pancreatic acinar cell damage, immune cell infiltration, and fatty changes, some acinar cells with normal histology and morphology persist. (*A*) H&E-stained whole pancreas at 1× magnification. (*B*) Local view of the H&E-stained pancreatic tissue at 20× magnification.

One observation that raises a question about the role of apoptosis in these models is our previously reported finding that only 1% to 2% of acinar cells are positive for tunnel staining at the time points tested.^16^ This finding could be interpreted as indicating that apoptosis is not a major pathway for cell death. More likely, the low level of apoptosis results from differing rates of cell injury among pancreatic acinar cells. We know that acinar cell mass decreases with time. Yet histologically normal acinar cells persist well into the second year of life (Figure 22). It remains unknow why there are differences in the time it takes for cell death of acinar cells to occur in our model. Supporting this possibility are observations from immunohistochemistry of acinar cells and single-cell RNA-seq that reveal subpopulations of acinar cells that differ in gene or protein expression particularly of zymogens.^43^, ^44^, ^45^, ^46^, ^47^, ^48^ Differences in the degree and rate of injury could also explain the complex milieu of pro- and anti-apoptotic regulators we observed. An alternate explanation is that other cell types such as immune cells in the pancreas contribute to the detected expression pattern.

Intriguingly, the amount of PNLIP T221M in the detergent-dependent insoluble fraction decreased with age, even as pathological changes in the pancreas progressed in mutant homozygous mice. Similarly, as seen in *Pnlip* T221M heterozygous mice, CPA1 N256K protein present in the detergent-insoluble fraction remains much lower in *Cpa1* N256K mice than that observed in *Pnlip* T221M homozygous mice, yet the disease progresses with age.^16^ We have speculated several explanations for this paradox. First, different populations of acinar cells may express varying levels of zymogens, including PNLIP and CPA1, a concept supported by recent studies of mRNA expression in single cells or nuclei isolated from the pancreas.^45^ In this case, acinar cells that express higher levels of zymogens, including PNLIP and CPA1, may be less able to restore homeostasis and succumb earlier in the course of the disease, whereas acinar cells expressing lower levels of zymogens may survive longer before succumbing to the continued stress of handling misfolded digestive enzymes. We observed a small but increased number of acinar cells undergoing cell death in both models at both 1 and 3 months. Second, there may be ongoing selection for pancreatic acinar cells that are more efficient in disposing of misfolded proteins, resulting in an acinar cell population with gradually decreasing accumulation of misfolded mutant digestive enzymes in our mouse models. However, our RNA-seq data on ERAD and ER-phagy, which shows decreased expression of most of the key components in these two pathways, contradicts this notion. Third, it is worth exploring whether misfolded digestive enzymes consume critical proteins such as BiP and other molecular chaperones, depleting their bioavailability in pancreatic acinar cells and therefore disturbing their critical functions, leading to cell injury. Fourth, once the inflammatory response starts, the increased cytokine production and infiltration of inflammatory cells may continue unchecked, leading to ongoing injury and death of acinar cells. Finally, a surprising finding for both models is that intrapancreatic trypsin levels were elevated.^16^^,^^22^ Although we believe that the increased levels of trypsin are more likely a byproduct of pancreatic injury or cell death rather than the driving force for the disease, further studies involving inhibition of intrapancreatic trypsin activity may help determine whether trypsin activation contributes to disease progression despite the decreased amount of misfolded protein.^49^ As bulk RNA-seq data has its limitations, further studies are needed to employ the more powerful single-cell (or single-nucleus) RNA-seq to precisely identify cell-specific transcriptomic signatures across different ages to dissect the disease mechanisms in our CP model.

A major weakness of the current pathway analysis lies in the technical limitations of total RNA-seq. First, it cannot identify the specific cell types responsible for the signal. Although acinar cells account for most cells in the pancreas, they likely do not account for all the differential gene expression described in this study. The other cell types in the pancreas may contribute. For instance, the infiltrating inflammatory cells will contribute to the inflammation and immune signatures detected in this analysis. Observations presented herein and in previous publications help identify the activated pathways in the acinar cells. Our earlier study of *Pnlip* T221M mice showed increased staining for markers of apoptosis and p53 activation in acinar cells.^16^ Second, total RNA-seq lacks the resolution depth offered by singe-cell or single-nuclei RNA-seq. Even in acinar cells, recent studies have demonstrated heterogeneity within acinar cells regarding the mass of zymogens expressed as just discussed above. Performing quality single-cell or single-nuclei RNA-seq analysis of mouse pancreatic tissue is challenging. It is difficult to obtain single acinar cell suspension from clusters of acini, especially in a stressed state, or prepare single-nuclei suspension due to severe RNA degradation associated with high levels of endogenous RNase present in the pancreatic tissues especially mouse pancreas.^50^ Further analysis of these pathways at the single-cell-level will provide more in-depth insights.

Importantly, TEM images of *Pnlip* T221M mice show dilated, disrupted mitochondria with loss of the outer membrane consistent with the changes reported during apoptosis.^51^ The TEM images confirmed our previous report of dilated ER, a marker of ER stress. TEM images of *Cpa1* N256K mice did not show the same mitochondrial changes, and ER dilation was minimal, consistent with the observation that the CP induced by the *CPA1* variant may not be as severe as that induced by the *PNLIP* variant.^22^ Examination of the differentially expressed mitochondrial genes and nuclear-encoded mitochondrial genes confirmed this difference between the two models. Pathways for abnormal mitochondrial and crista morphology and intrinsic apoptosis were upregulated only in *Pnlip* T221M mice. *Pink1* is a component of these pathways, and its expression was upregulated as confirmed by qPCR in *Pnlip* T221M mice. PINK1 is critical for mitochondrial quality control and marks damaged mitochondria for degradation.^52^^,^^53^

## Conclusion

In conclusion, our comprehensive comparative study of *Cpa1* N256K and *Pnlip* T221M mice demonstrates that both mouse models develop CP through similar mechanisms, although with slightly different progression and severity. Examination of differential expression and pathway analysis shows that both intrinsic and extrinsic apoptosis are activated early in the course of the disease. These pathways are triggered through multiple pathways. Thus, it will be difficult to target these pathways for therapy development. Approaches to reducing the burden of misfolded protein through decreased expression of the variant allele, increased efficiency of folding, or increased rates of degradation will be required. Both models are valuable for understanding the disease mechanism associated with protein misfolding and for developing and testing therapies to prevent and treat this subtype of CP.

## Materials and Methods

### Mouse Models and Their Care and Use

The *Cpa1* N256K mouse model was originally generated by Dr MiklósSahin-Tóth at Boston University as previously described.^22^ These mice were transferred from Dr Sahin-Toth’s group currently at the University of California, Los Angeles to Washington University in St Louis for this study. The reference accession numbers for the *Mus musculus Cpa1* genomic DNA and cDNA sequences are NC_000072.7 and NM_025350.4, respectively. Our group previously reported the generation of the *Pnlip* T221M mouse model.^16^ The reference accession numbers for *Mus musculus Pnlip* genomic DNA and cDNA sequences are NC_000085.7 and NM_026925.4, respectively. Both mouse models were generated and maintained on the C57BL/6N genetic background (Charles River Laboratory).

The mice were housed in the same room at Washington University following the guidelines and policies of the Institutional Animal Care and Use Committee (IACUC) (protocol ID: 22-0356). Mouse husbandry was conducted by the Mouse Genetics Core personnel, and the animals were maintained under a standard 12-hour light/dark cycle and provided ad libitum access to water and regular chow. Breeding was performed using heterozygous males and females for each strain, and pups were weaned at 21 days of age. Homozygous, heterozygous, and WT mice from the *Cpa1* N256K and *Pnlip* T221M strains were analyzed separately by sex at the indicated ages. Genotyping for both strains was performed commercially by Transnetyx using validated qPCR methods.

### Collection and Processing of Mouse Samples

Mice were sacrificed at the indicated ages using CO_2_ euthanasia followed by cervical dislocation, typically performed in the early afternoon. After euthanasia, the body weight of each mouse was measured, and the entire pancreas was excised and weighed. For histological staining, the pancreas was fixed in 10% neutral buffered formalin at room temperature with gentle agitation for 24 to 48 hours. For biochemical and molecular assays, the entire pancreas was immediately frozen in liquid nitrogen and ground into a fine powder under liquid nitrogen. Aliquots of pancreas powder were stored at −80°C for subsequent analysis.

### Histology and Immunohistochemistry

Formalin-fixed pancreas tissue was processed, embedded in paraffin, and sectioned at a thickness of 5 μm for staining. Both histology and immunohistochemistry were performed by HistoWiz or at the Digestive Disease Research Center Core (DDRCC) at Washington University in St. Louis. For histology, the sections were stained using H&E, and Masson’s trichrome blue staining was used to assess pancreatic fibrosis. For immunohistochemistry, staining was performed using standard protocols as previously described.^16^ The primary antibodies used in this study were as follows: a rabbit monoclonal F4/80 antibody (1:100; 70076, Cell Signaling Technology); a rabbit monoclonal CD3 antibody (1:100; 16669, Abcam); a rabbit polyclonal CD45 antibody (1:2000; 10558, Abcam); a rabbit polyclonal myeloperoxidase (MPO) antibody (1:50; 9535, Abcam); a rabbit polyclonal p53 antibody (1:200; NCL-L-p53-CM5p, Leica Biosystems); a rabbit monoclonal p21 antibody (1:500; 188224, Abcam); a rabbit polyclonal Ki67 antibody (1:800; 15580, Abcam); a rabbit polyclonal phospho-histone H3 Ser10 (p-HH3) antibody (1:1200; 5176, Abcam); a rabbit monoclonal phospho-histone H2A.X Ser139 (p-γH2A.X) antibody (1:800; 9718, Cell Signaling Technology); a rabbit polyclonal CC3 antibody (1:300; 9661, Cell Signaling Technology). Bond Polymer Refine Detection (Leica Biosystems) was utilized according to the manufacturer’s protocol. After staining, sections were dehydrated and cover-slipped using a TissueTek-Prisma and Coverslipper (Sakura). Whole-slide scanning at 40× magnification was performed using an Aperio AT2 (Leica Biosystems). The slides were viewed and analyzed using Aperio ImageScope software (Leica Biosystems).

### Image Quantification

Whole-slide scanned images of pancreas histology and immunohistochemistry were used to quantify pancreatic acinar cell loss, fibrosis, and specific marker-positive cell populations, following the detailed protocol described previously.^16^

Briefly, pancreatic acinar cell loss, fibrosis, and macrophages were quantified using ImageJ software on pancreas sections stained by H&E, Masson’s trichrome blue, and immunohistochemistry for the F4/80 marker, respectively. The software was used to highlight areas of nonacinar cell annotations, and acinar cell loss was measured as a percentage of the total area of pancreatic tissue. Fibrotic tissue areas, stained areas of fibrotic tissue, were highlighted and measured as a percentage of the total area to calculate fibrosis. Macrophages were quantified similarly, with highlighted portions representing macrophages measured as a percentage of the total area.

The quantification of other immunohistochemistry markers on immunostained mouse pancreatic sections was carried out using the QuPath software, following previously detailed protocols.^16^ The immunohistochemistry markers included CD3, CD45, MPO, p53, p21, Ki67, p-HH3, p-γH2A.X, and CC3. 4′,6-diamidino-2-phenylindole (DAPI) was used to counterstain nuclei. Using the software’s positive cell detection feature, the number of cells or nuclei positive for each marker and the number of DAPI- stained nuclei (representing total cell numbers) were counted. The quantification of each immunohistochemistry stain was expressed as the percentage of marker-positive cells or nuclei relative to the total cells.

### TEM

Sample fixation, processing, and image acquisition for TEM were carried out as previously described.^16^ Small pieces (1–3 mm^3^) of mouse pancreatic tissue were initially fixed in a solution containing 2% paraformaldehyde and 2.5% glutaraldehyde in 0.15 M cacodylate buffer with 2 mM CaCl_2_, pH 7.4. The fixed tissues were then submitted to the Washington University Center for Cellular Imaging (WUCCI) for further processing and image acquisition.

Briefly, the tissues underwent secondary fixation in 2% osmium tetroxide/1.5% potassium ferrocyanide in cacodylate buffer, followed by staining in an aqueous solution of 1% uranyl acetate. The stained samples were subsequently dehydrated through a graded acetone series (50%, 70%, 90%, and 100% × 3) and infiltrated with Spurr’s resin. After curing at 60°C for 72 hours, 70-nm thin sections were cut from the resin block and stained with uranyl acetate and Reynold’s lead. Finally, images were acquired using a JEM-1400 Plus transmission electron microscope (JEOL Inc.) operating at 120 keV.

### Immunoblotting

The preparation and processing of whole cell lysates and detergent-insoluble fractions from mouse pancreatic samples were performed following our previously detailed protocol.^16^ Briefly, 20 to 30 mg of mouse pancreas powder was homogenized in RIPA buffer (R0278, Sigma) supplemented with protease and phosphatase inhibitor cocktails, using a ratio of 30 μL RIPA buffer per mg of pancreas powder. The homogenate was then sonicated to ensure complete protein extraction. A 500-μL portion of the homogenate was used for further processing. Of this, 75 μL was mixed with an equal volume of 2× Laemmli buffer, boiled at 95°C for 5 minutes, and briefly sonicated. This preparation was designated as the whole cell lysate. The remaining 425 μL of the crude protein lysate was centrifuged at 16,000 g, and the resulting pellet was resuspended in 100 μL of 1× Laemmli buffer (a mixture of equal volumes of 2× Laemmli buffer and RIPA) followed by sonication. This final suspension was designated as the insoluble fraction. Equal volumes of the whole cell lysate and the insoluble fraction protein lysates were then mixed with an equal volume of 2× SDS Sample Buffer (1610737, Bio-Rad) supplemented with 1.14 M of β-mercaptoethanol and boiled at 95°C for 5 minutes. For each sample type, 30 μL of the processed protein lysates per lane were loaded on 4% to 15% Mini-PROTEAN TGX pre-cast gels (4561083, Bio-Rad) for gel electrophoresis and transferred onto Immobilon PVDF FL membranes (IPFL00010, Millipore). The membranes were first blocked for a minimum of 60 minutes before incubation with a primary antibody for 60 minutes at room temperature or overnight at 4°C. The primary antibodies used for immunoblotting included a goat polyclonal CPA1 antibody (1:3000; AF2765, R&D Systems), a mouse monoclonal PNLIP antibody (1:2000; sc393085, Santa Cruz Biotechnology), and a rat monoclonal α-tubulin antibody against (1:1000; SC53029, Santa Cruz Biotechnology). Alpha-tubulin was used as loading control. This was followed by incubation with a corresponding secondary antibody for 60 minutes at room temperature. The secondary antibodies included an IRDye 800CW donkey anti-goat IgG (926-32214), an IRDye 800CW goat anti-mouse IgG (926-32210), and an IRDye 680RD goat anti-rat IgG (926-68076), all from LI-COR used at 1:10,000 dilution. Blot images were captured using a ChemiDoc MP system, and band densitometry was determined using Image Lab software (Bio-Rad).

### RNA Isolation, Reverse Transcription, and qPCR

Total RNA from mouse pancreatic tissue was isolated using the RNeasy Plus Mini Kit (74134, Qiagen), and subsequent reverse transcription was carried out according to the protocols previously described.^16^ The RNA integrity was assessed by gel electrophoresis containing 1% bleach and visualized by ethidium bromide staining.^54^ Reverse transcription was carried out using the QuantiTect Reverse Transcription Kit (205311, Qiagen). The synthesized complementary DNA (cDNA) was diluted 10-fold and served as the template for qPCR. The qPCR analysis was performed on a QuantStudio 3 System using TaqMan Fast Advanced Master Mix (4444964) and gene-specific probes listed in Table 9. The machine, reagents, and probes were all obtained from Thermo Fisher Scientific. Cycle threshold (Ct) values obtained from the qPCR assays were analyzed using the ΔΔCt method. The expression values of the gene of interest in mutant *Cpa1* N256K or *Pnlip* T221M homozygous mice were normalized to 18S rRNA levels and then compared with those in WT mice. This allowed for the calculation of the fold change in the expression of the gene of interest in the mutant mice relative to the WT group.

**Table 9:** TaqMan Gene Expression Assay Probe List

Gene symbol	Assay ID
*Apaf1*	Mm01223701_m1
*Atf3*	Mm00476032_m1
*Atf4*	Mm00515325_g1
*Bak1*	Mm00432045_m1
*Bbc3*	Mm00519268_m1
*Bcl2l11*	Mm00437796_m1
*BiP (Hspa5)*	Mm00517691_m1
*Casp3*	Mm01195085_m1
*Casp7*	Mm00432322_m1
*Casp9*	Mm00516563_m1
*CHOP (Ddit3)*	Mm01135937_g1
*Ddias*	Mm00546937_m1
*Fas*	Mm01204974_m1
*Fgf21*	Mm00840165_g1
*Gadd45a*	Mm00432802_m1
*Hamp2*	Mm00842044_g1
*Hspa1a*	Mm01159846_s1
*Hspa1b*	Mm03038954_s1
*p21 (Cdkn1a)*	Mm04205640_g1
*Rn18s, Rn45s*	Mm03928990_g1
*Tnfrsf10b*	Mm00457866_m1
*Tnfsf10*	Mm01283606_m1
*Trp53*	Mm01731290_g1
*Xaf1*	Mm01248390_m1

### Total RNA-Seq Analysis

Total RNA-seq was performed by The Genome Access Technology Center (GTAC) at the McDonnell Genome Institute (MGI) at Washington University in St. Louis. Five samples each from WT, *Cpa1* N256K homozygous and *Pnlip* T221M homozygous male mice at 1 and 3 months were analyzed, totaling 60 samples. Total RNA integrity was determined using Agilent Bioanalyzer or 4200 Tapestation. The RIN values were 9.07 (mean) ± 0.84 (SD), ranging from 6.7 to 10. Library preparation was performed with 5 to 10 ug of total RNA with a RIN score greater than 8.0. Ribosomal RNA was removed by poly-A selection using Oligo-dT beads (mRNA Direct kit, Life Technologies). mRNA was then fragmented in reverse transcriptase buffer and heated at 94°C for 8 minutes, and mRNA was reverse transcribed to yield cDNA using SuperScript III RT enzyme (Life Technologies) and random hexamers. A second strand reaction was performed to yield ds-cDNA. cDNA was blunt ended, had an A base added to the 3' ends, and then had Illumina sequencing adapters ligated to the ends. Ligated fragments were then amplified for 12 to 15 cycles using primers incorporating unique dual index tags. Fragments were sequenced on an Illumina NovaSeq-6000 using paired end reads extending 150 bases with a targeted number of reads set at 30 million per sample. RNA-seq reads were then aligned and quantitated to the Ensembl release 101 primary assembly with an Illumina DRAGEN Bio-IT on-premise server running version 3.9.3-8 software. Gene expression was compared across samples by linear modeling (limma/voom).^55^ Differential expression analysis was then performed to analyze for differences between conditions, and the results were filtered for only those genes with Benjamini-Hochberg FDR adjusted *P* values less than or equal to .05. Pathway analysis was performed with Generally Applicable Gene-Set Enrichment (GAGE) or Gene Set Enrichment Analysis (GSEA) using Mouse MSigDB v2023.1.^56^^,^^57^ Pathway analysis of GSEA leading-edge genes was performed with Enrichr.^58^, ^59^, ^60^ Cluster analysis was done with ShinyGO.^61^

### Statistical Analysis

One- or 2-way analysis of variance (ANOVA) with Tukey’s multiple comparisons test was performed using GraphPad Prism 10, as specified in the figure legends. A *P* value of < .05 was considered statistically significant.
